# Local Criteria for Triangulating General Manifolds

**DOI:** 10.1007/s00454-022-00431-7

**Published:** 2022-09-30

**Authors:** Jean-Daniel Boissonnat, Ramsay Dyer, Arijit Ghosh, Mathijs Wintraecken

**Affiliations:** 1 INRIA Sophia Antipolis, Université Côte d’Azur, Sophia Antipolis, France; 2 Indian Statistical Institute, Kolkata, India; 3 IST Austria, Klosterneuburg, Austria

**Keywords:** Triangulating/Meshing, Triangulation criteria, Local coordinate charts, Manifolds, 57Q15, 68U05, 57R05

## Abstract

We present criteria for establishing a triangulation of a manifold. Given a manifold *M*, a simplicial complex ${\mathscr {A}}$, and a map *H* from the underlying space of ${\mathscr {A}}$ to *M*, our criteria are presented in local coordinate charts for *M*, and ensure that *H* is a homeomorphism. These criteria do not require a differentiable structure, or even an explicit metric on *M*. No Delaunay property of ${\mathscr {A}}$ is assumed. The result provides a triangulation guarantee for algorithms that construct a simplicial complex by working in local coordinate patches. Because the criteria are easily verified in such a setting, they are expected to be of general use.

## Introduction

A *triangulation* of a manifold $M$ is a homeomorphism $H:|{\mathscr {A}}| \rightarrow M$, where ${\mathscr {A}}$ is a simplicial complex, and $|{\mathscr {A}}|$ is its underlying topological space. If such a homeomorphism exists, we say that ${\mathscr {A}}$ *triangulates* $M$.

The purpose of this paper is to present criteria which ensure that a candidate map *H* is indeed a homeomorphism. This work is motivated by earlier investigations into the problem of algorithmically constructing a complex that triangulates a given manifold [5, 8]. It complements and is closely related to recent work that investigates a particular natural example of such a map [16].

In the motivating algorithmic setting, we are given a compact manifold $M$, and a manifold simplicial complex ${\mathscr {A}}$ is constructed by working locally in Euclidean coordinate charts. Here we lay out criteria, based on local properties that arise naturally in the construction of ${\mathscr {A}}$, that guarantee that *H* is a homeomorphism. These criteria, which are summarized in Theorem 2.17, are based on metric properties of *H* within “compatible” coordinate charts (Definition 2.4). That is, we make sure the linearization of *H* inside each coordinate chart is an embedding. If simplices are sufficiently thick and the original map is similar enough to the linearized one, this guarantees that *H* is a local homeomorphism. One needs an additional technical criterion to make sure that it is a homeomorphism rather than a covering map. The Euclidean metric in the local coordinate chart is central to the analysis, but no explicit metric on $|{\mathscr {A}}|$ or $M$ is involved, and no explicit assumption of differentiability is required of *H* or $M$. However, our only examples that meet the required local criteria are in the differentiable setting. We do not know whether or not our criteria for homeomorphism implicitly imply that $M$ admits a differentiable structure. They do imply that ${\mathscr {A}}$ is piecewise linear (admits an atlas with piecewise linear transition functions).

### Relation to Other Work

The first demonstrations that differentiable manifolds *can* always be triangulated were constructive. Cairns [13] used coordinate charts to cover the manifold with embeddings of patches of Euclidean triangulations. He showed that if the complexes were sufficiently refined the embedding maps could be perturbed such that they remain embeddings and the images of simplices coincide where patches overlap. A global homeomorphic complex is obtained by identifying simplices with the same image. The technique was later refined and extended [21, 23], but it is not easily adapted to provide triangulation guarantees for complexes constructed by other algorithms.

#### Submanifolds of ${\mathbb {R}}^N$

An alternative approach was developed by Whitney [9, 24] using his result that a manifold can be embedded into Euclidean space. A complex is constructed via a process involving the intersection of the manifold with a fine Cartesian grid in the ambient space, and it is shown that the *closest-point projection map*, which takes a point in the complex to its unique closest point in the manifold, is a homeomorphism. The argument is entwined with this specific construction, and is not easily adapted to other settings.

More recently, Edelsbrunner and Shah [18] (using general results on CW-complexes, see for example [20]) defined the restricted Delaunay complex of a subset *M* of Euclidean space as the nerve of the Voronoi diagram on *M* when the ambient Euclidean metric is used. They showed that if *M* is a compact manifold, then the restricted Delaunay complex is homeomorphic to *M* when the Voronoi diagram satisfies the *closed ball property*: Voronoi faces are closed topological balls of the appropriate dimension.

Using the closed ball property, Amenta and Bern [1] demonstrated a specific sampling density that is sufficient to guarantee that the restricted Delaunay complex triangulates the surface. However, since the complex constructed by their reconstruction algorithm cannot be guaranteed to be exactly the restricted Delaunay complex, a new argument establishing homeomorphism was developed, together with a simplified version of the algorithm [2].

Although it was established in the context of restricted Delaunay triangulations, the closed ball property is an elegant topological result that applies in more general contexts. For example, it has been used to establish conditions for intrinsic Delaunay triangulations of surfaces [17], and Cheng et al. [14] have indicated how it can be applied for establishing weighted restricted Delaunay triangulations of smooth submanifolds of arbitrary dimension in Euclidean space.

However, the closed ball property is only applicable to Delaunay-like complexes that can be realized as the nerve of some kind of Voronoi diagram on the manifold. Thus, for example, it does not necessarily apply to the tangential Delaunay complex constructed by Boissonnat and Ghosh [8]. Secondly, even when a Delaunay-like complex is being constructed, it can be difficult to directly verify the properties of the associated Voronoi structure. The closed ball property does not give us sampling criteria and conditions on the complex under construction. A third deficiency of the closed ball property is that, although it can establish that a complex ${\mathscr {A}}$ triangulates the manifold $M$, it does not provide a specific triangulation $H:|{\mathscr {A}}| \rightarrow M$. Such a correspondence is required to compare *geometric* properties of $|{\mathscr {A}}|$ and $M$.

In [8] Whitney’s argument was adapted to demonstrate that the closest-point projection maps the tangential Delaunay complex homeomorphically onto the original manifold. The argument is intricate, and like Whitney’s, is tailored to the specific complex under consideration. In contrast, the result of [2], especially in the formulation presented by Boissonnat and Oudot [11], guarantees a triangulation of a surface by any complex which satisfies a few easily verifiable properties. However, the argument only holds for surfaces in ${\mathbb {R}}^3$.

#### Riemannian Manifolds

If a set of vertices is contained within a sufficiently small neighbourhood on a Riemannian manifold, barycentric coordinates can be defined. So there is a natural map from a Euclidean simplex of the appropriate dimension to the manifold, assuming a correspondence between the vertices of the simplex and those on the manifold. Thus when a complex ${\mathscr {A}}$ is appropriately defined with vertices on a Riemannian manifold $M$, there is a natural *barycentric coordinate map* $|{\mathscr {A}}| \rightarrow M$. In [16], conditions are presented which guarantee that this map is a triangulation. Although this map can also be applied to submanifolds of Euclidean space, it is often not easy to compute because it boils down to finding geodesics, which is far more complicated than computing closest points projections. This is perhaps the main reason why we have developed the general approach of this paper. When revisiting the result for Riemannian manifolds we also generalize to smooth maps and simplify the exposition.

### Outline

The general triangulation criteria (Theorem 2.17) are given in Sect. 2, without explicitly defining the map *H*. In Sect. 3, Theorem 2.17 is applied to the specific case where $M\subset {\mathbb {R}}^N$, and *H* is the projection to the closest-point on $M$. This is a refinement of the argument presented in [8], also correcting an error. Section 4 explores the differentiable setting, which is a generalization of the setting where $M$ is a Riemannian manifold, and plugs a gap in [16]. In Appendix A, we discuss some lemmas on general distortion maps. In Appendix B we review degree theory and discuss the link with Whitney’s work.

## The Homeomorphism Criteria

We assume that ${\mathscr {A}}$ and $M$ are both compact manifolds of dimension *m*, without boundary, and we have a map $H:|{\mathscr {A}}| \rightarrow M$ that we wish to demonstrate is a homeomorphism. We first show that *H* is a covering map, i.e., every $y\in M$ admits an open neighbourhood $U_y$ such that $H^{-1}(y)$ is a disjoint union of open sets each of which is mapped homeomorphically onto $U_y$ by *H*. In our setting it is sufficient to establish that *H* is a local homeomorphism whose image touches all components of $M$: Brouwer’s invariance of domain then ensures that *H* is surjective, and, since $|{\mathscr {A}}|$ is compact, has the covering map property.

### Notation 2.1

(*simplices and stars*)   In this section, a simplex $\varvec{\sigma }$ will always be a *full simplex*: a closed Euclidean simplex, specified by a set of vertices together with all the points with nonnegative barycentric coordinates. The *relative interior* of $\varvec{\sigma }$ is the topological interior of $\varvec{\sigma }$ considered as a subspace of its affine hull, and is denoted by ${{\,\mathrm{relint}\,}}(\varvec{\sigma })$. If $\varvec{\sigma }$ is a simplex of ${\mathscr {A}}$, the subcomplex consisting of all simplices that have $\varvec{\sigma }$ as a face, together with the faces of these simplices, is called the *star* of $\varvec{\sigma }$, denoted by ${\underline{\mathrm {St}}}(\varvec{\sigma })$; the star of a vertex *p* is ${\underline{\mathrm {St}}}(p)$.

We also sometimes use the *open star* $\mathrm {st}(\varvec{\sigma })$ of a simplex $\varvec{\sigma }\in {\mathscr {C}}$. This is the union of the relative interiors of the simplices in ${\mathscr {C}}$ that have $\varvec{\sigma }$ as a face: $\mathrm {st}(\varvec{\sigma }) = \bigcup _{\varvec{\tau }\supseteq \varvec{\sigma }}{{\,\mathrm{relint}\,}}(\varvec{\tau })$. It is an open set in $|{\mathscr {C}}|$, and it is open in ${\mathbb {R}}^m$ if $\varvec{\sigma }\not \in \partial {{\mathscr {C}}}$.

### Notation 2.2

(*topology*)   If $A \subseteq {\mathbb {R}}^m$, then the topological closure, interior, and boundary of *A* are denoted respectively by $\overline{A}$, ${{\,\mathrm{int}\,}}(A)$, and $\partial {A} =\overline{A} \setminus {{\,\mathrm{int}\,}}(A)$. We denote by $B_{{\mathbb {R}}^m}(c,r)$ the open ball in ${\mathbb {R}}^m$ of radius *r* and centre *c*.

### Notation 2.3

(*linear algebra*)  The Euclidean norm of $v\in {\mathbb {R}}^m$ is denoted by $\Vert v\Vert$, and $\Vert A\Vert =\sup _{\Vert x\Vert =1}\Vert Ax\Vert$ denotes the operator norm of the linear operator *A*.

We will work in local coordinate charts. To any given map $G:|{\mathscr {C}}|\rightarrow {\mathbb {R}}^m$, where ${\mathscr {C}}$ is a simplicial complex, we associate a piecewise linear map ${\widehat{G}}$ that agrees with *G* on the vertices of ${\mathscr {C}}$, and maps $x\in \varvec{\sigma }\in {\mathscr {C}}$ to the point with the same barycentric coordinates with respect to the images of the vertices. The map ${\widehat{G}}$ is called the *secant map* of *G* with respect to ${\mathscr {C}}$.

The following definition provides the framework within which we will work (see diagram (2)).

### Definition 2.4

(*compatible atlases*)   We say that $|{\mathscr {A}}|$ and $M$ have *compatible atlases* for $H:|{\mathscr {A}}| \rightarrow M$ if:

(i) There is a coordinate atlas $\{(U_p, \phi _p)\}_{p\in {\mathscr {P}}}$ for $M$, where the index set ${\mathscr {P}}$ is the set of vertices of ${\mathscr {A}}$ and each set $U_p$ is connected.
(ii) For each $p\in {\mathscr {P}}$, there is a subcomplex ${\widetilde{{\mathscr {C}}}}_p$ of ${\mathscr {A}}$ that contains ${\underline{\mathrm {St}}}(p)$ and $H(|{\widetilde{{\mathscr {C}}}}_p|) \subset U_p$. Also, the secant map of $\varPhi _p := \phi _p \circ H|_{|{\widetilde{{\mathscr {C}}}}_p|}$ defines a piecewise linear embedding of $|{\widetilde{{\mathscr {C}}}}_p|$ into ${\mathbb {R}}^m$. We denote this secant map by ${\widehat{\varPhi }}_p$. By definition, ${\widehat{\varPhi }}_p$ preserves the barycentric coordinates within each simplex, and thus the collection $\{({\widetilde{{\mathscr {C}}}}_p,{\widehat{\varPhi }}_p)\}_{p\in {\mathscr {P}}}$ provides a piecewise linear atlas for ${\mathscr {A}}$.

### Remark 2.5

The requirement in Definition 2.4 that the local patches $U_p$ be connected implies that on each connected component $M'$ of *M*, there is a $p\in {\mathscr {P}}$ such that $H(p)\in M'$.

We let ${\mathscr {C}}_p = {\widehat{\varPhi }}_p({\widetilde{{\mathscr {C}}}}_p)$, and we will work within the compatible local coordinate charts. Thus we are studying a map of the form

$$\begin{aligned} F_p :|{\mathscr {C}}_p| \subset {\mathbb {R}}^m\rightarrow {\mathbb {R}}^m, \end{aligned}$$

where ${\mathscr {C}}_p$ is an *m*-manifold complex with boundary embedded in ${\mathbb {R}}^m$, and

1 $$\begin{aligned} F_p = \phi _p \circ H \circ {\widehat{\varPhi }}_p^{-1}, \end{aligned}$$

as shown in the following diagram: 
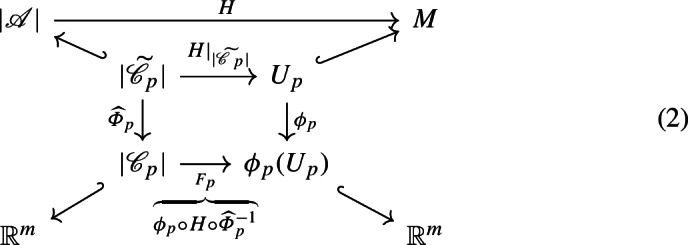


We will focus on the map $F_p$, which can be considered as a local realisation of $H|_{|{\widetilde{{\mathscr {C}}}}_p|}$. By construction, $F_p$ leaves the vertices of ${\mathscr {C}}_p$ fixed: if $q \in {\mathbb {R}}^m$ is a vertex of ${\mathscr {C}}_p$, then $F_p(q)=q$, since ${\widehat{\varPhi }}_p$ coincides with $\phi _p \circ H$ on vertices.

### Remark 2.6

The setting described here encapsulates the one in the tangential complex work [8] and the one in the Riemannian triangulation paper [16]. In [8] one locally constructs a (weighted) Delaunay triangulation in the tangent space $T_pM$. This gives us the local patch ${\mathscr {C}}_p$ (the star of *p* in $T_pM$). The vertices of the constructed complex actually lie on $M$, and we recognise ${\mathscr {C}}_p$ as the orthogonal projection ${\widehat{\varPhi }}_p$ of the corresponding complex ${\widetilde{{\mathscr {C}}}}_p$ with vertices on $M$. In this context, the homeomorphism *H* that we are trying to establish is given by the closest-point projection map onto $M$, restricted to $|{\mathscr {A}}|$. Now we are going to work in local coordinate charts, given at each vertex $p \in {\mathscr {P}}$ by the orthogonal projection $\phi _p$ of some neighbourhood of *p*, $U_p \subset M$ into $T_pM$. We recognise that ${\widehat{\varPhi }}_p$ really does coincide with the secant map of $\phi _p \circ H|_{|{\widetilde{{\mathscr {C}}}}_p|}$. In [16] the simplices are mapped to the Riemannian manifold using Riemannian centres of mass. In the Riemannian setting the coordinate charts are the natural concepts to work with from the outset.

### Local Homeomorphism

Our goal is to ensure that there is some open $V_p \subset |{\mathscr {C}}_p|$ such that $F_p|_{V_p}$ is an embedding and that the sets ${\widetilde{V}}_p = {\widehat{\varPhi }}_p^{-1}(V_p)$ are sufficiently large to cover $|{\mathscr {A}}|$. This will imply that *H* is a local homeomorphism. Indeed, if $V_p$ is embedded by $F_p$, then ${\widetilde{V}}_p$ is embedded by $H|_{{\widetilde{V}}_p} = \phi _p^{-1}\circ F_p \circ {\widehat{\varPhi }}_p|_{{\widetilde{V}}_p}$, since $\phi _p$ and ${\widehat{\varPhi }}_p$ are both embeddings. Since $|{\mathscr {A}}|$ is compact, Brouwer’s invariance of domain, together with Remark 2.5, implies that *H* is surjective, and a covering map. It will only remain to ensure that *H* is also injective.

We assume that we are given (i.e., we can establish by context-dependent means) a couple of properties of $F_p$. We assume that it is *simplexwise positive*, which means that it is continuous, and its restriction to any *m*-simplex in ${\underline{\mathrm {St}}}({\hat{p}})$ is an orientation preserving topological embedding. As discussed in Appendix B, we say that $F_p$ preserves the orientation of an *m*-simplex $\varvec{\sigma }\subset {\mathbb {R}}^m$ if $F_p|_{\varvec{\sigma }}$ has degree 1 at any point in the image of the interior of $\varvec{\sigma }$, i.e., $\deg {(F_p,{{\,\mathrm{int}\,}}(\varvec{\sigma }),y)}=1$ for $y\in F_p({{\,\mathrm{int}\,}}(\varvec{\sigma }))$. (The other assumption we make is that $F_p$, when restricted to an *m*-simplex does not distort distances very much, as discussed below.)

The local homeomorphism demonstration is based on Lemma 2.7 below, which is a particular case of an observation made by Whitney [24, Appx. II, Lem. 15a]. Whitney demonstrated a more general result from elementary first principles. The proof we give here is the same as Whitney’s, except that we exploit elementary degree theory, as discussed in Appendix B, in order to avoid the differentiability assumptions Whitney made.

In the statement of the lemma, ${\mathscr {C}}^{m-1}$ refers to the $(m{-}1)$*-skeleton* of ${\mathscr {C}}$: the subcomplex consisting of simplices of dimension less than or equal to $m-1$. When $|{\mathscr {C}}|$ is a manifold with boundary, as in the lemma, then $\partial {\mathscr {C}}$ is the subcomplex containing all $(m-1)$-simplices that are the face of a single *m*-simplex, together with the faces of these simplices.

#### Lemma 2.7

(simplexwise positive embedding)   Assume ${\mathscr {C}}$ is an oriented *m*-manifold finite simplicial complex with boundary embedded in ${\mathbb {R}}^m$. Let $F :|{\mathscr {C}}| \rightarrow {\mathbb {R}}^m$ be simplexwise positive in ${\mathscr {C}}$. Suppose $V \subset |{\mathscr {C}}|$ is a connected open set such that $F(V) \cap F(|\partial {{\mathscr {C}}}|) =\emptyset$. If there is a $y\in F(V) \setminus F(|{\mathscr {C}}^{m-1}|)$ such that $F^{-1}(y)$ is a single point, then the restriction of *F* to *V* is a topological embedding.

#### Proof

Notice that the topological boundary of $|{\mathscr {C}}| \subset {\mathbb {R}}^m$ is equal to the carrier of the boundary complex (see, e.g., [4, Lems. 3.6 and 3.7]):

$$\begin{aligned} \partial {|{\mathscr {C}}|} = |\partial {{\mathscr {C}}}|. \end{aligned}$$

Let $\varOmega = |{\mathscr {C}}| \setminus |\partial {{\mathscr {C}}}|$. Since *F* is simplexwise positive, and *F*(*V*) lies within a connected component of ${\mathbb {R}}^m \setminus F(\partial \varOmega )$, the fact that $F^{-1}(y)$ is a single point implies that $F^{-1}(w)$ is a single point for any $w \in F(V) \setminus F(|C^{m-1}|)$ (Lemma B.9). We need to show that *F* is also injective on $V \cap |{\mathscr {C}}^{m-1}|$.

We now show that $F(\mathrm {st}(\varvec{\sigma }))$ is open for any $\varvec{\sigma }\in {\mathscr {C}}^{m-1} \setminus \partial {{\mathscr {C}}}$, where $\mathrm {st}(\varvec{\sigma })$ is the open star of $\varvec{\sigma }$, defined in Notation 2.1. Suppose $x \in {{\,\mathrm{relint}\,}}(\varvec{\tau })$ for some $\varvec{\tau }\in {\mathscr {C}}\setminus \partial {{\mathscr {C}}}$. Since *F* is injective when restricted to any simplex, we can find a sufficiently small open (in ${\mathbb {R}}^m$) neighbourhood *U* of *F*(*x*) such that $U \cap F(\partial {\mathrm {st}(\varvec{\tau })}) = \emptyset$. Since the closure of the open star is equal to the carrier of our usual star,

$$\begin{aligned} \overline{\mathrm {st}(\varvec{\tau })} = |{\underline{\mathrm {St}}}(\varvec{\tau })|, \end{aligned}$$

Lemma B.9 implies that every point in $U \setminus F(|{\underline{\mathrm {St}}}(\varvec{\tau })^{m-1}|)$ has the same number of points in its preimage. By the injectivity of *F* restricted to *m*-simplices, this number must be greater than zero for points near *F*(*x*). It follows that $U \subseteq F(\mathrm {st}(\varvec{\tau }))$.

If $x \in \mathrm {st}(\varvec{\sigma })$, then $x \in {{\,\mathrm{relint}\,}}(\varvec{\tau })$ for some $\varvec{\tau }\in {\mathscr {C}}\setminus \partial {{\mathscr {C}}}$ that has $\varvec{\sigma }$ as a face. Since $\mathrm {st}(\varvec{\tau }) \subseteq \mathrm {st}(\varvec{\sigma })$, we have $U \subseteq F(\mathrm {st}(\varvec{\sigma }))$, and we conclude that $F(\mathrm {st}(\varvec{\sigma }))$ is open.

Now, to see that *F* is injective on $|{\mathscr {C}}^{m-1}| \cap V$, suppose to the contrary that $w,z \in |{\mathscr {C}}^{m-1}| \cap V$ are two distinct points such that $F(w)=F(z)$. Since *F* is injective on each simplex, there are distinct simplices $\varvec{\sigma }, \varvec{\tau }$ such that $w \in {{\,\mathrm{relint}\,}}(\varvec{\sigma })$ and $z \in {{\,\mathrm{relint}\,}}(\varvec{\tau })$. So there is an open neighbourhood *U* of $F(w)=F(z)$ that is contained in $F(\mathrm {st}(\varvec{\sigma })) \cap F(\mathrm {st}(\varvec{\tau }))$.

We must have $\mathrm {st}(\varvec{\sigma }) \cap \mathrm {st}(\varvec{\tau }) = \emptyset$, because if $x\in \mathrm {st}(\varvec{\sigma })\cap \mathrm {st}(\varvec{\tau })$, then $x \in {{\,\mathrm{relint}\,}}(\varvec{\mu })$ for some $\varvec{\mu }$ that has both $\varvec{\sigma }$ and $\varvec{\tau }$ as faces. But this means that both *w* and *z* belong to $\varvec{\mu }$, contradicting the injectivity of $F|_{\varvec{\mu }}$. It follows that points in the nonempty set $U \setminus |{\mathscr {C}}^{m-1}|$ have at least two points in their preimage, a contradiction. Thus $F|_V$ is injective, and therefore, by Brouwer’s invariance of domain, it is an embedding. $\square$

Our strategy for employing Lemma 2.7 is to demand that the restriction of $F_p$ to any *m*-simplex has low metric distortion, and use this fact to ensure that the image of $V_p\subset |{\mathscr {C}}_p|$ is not intersected by the image of the boundary of $|{\mathscr {C}}_p|$, i.e., we will establish that $F_p(V_p)\cap F_p(|\partial {{\mathscr {C}}_p}|)=\emptyset$. We need to also establish that there is a point *y* in $F_p(V_p) \setminus F_p(|{\mathscr {C}}_p^{m-1}|)$ such that $F^{-1}(y)$ is a single point. The metric distortion bound will help us here as well.

#### Definition 2.8

($\xi$-*distortion map*)  A map $F:U\subset {\mathbb {R}}^m\rightarrow {\mathbb {R}}^m$ is a $\xi$*-distortion map* if for all $x,y \in U$ we have

3 $$\begin{aligned} \bigl |\Vert F(x)-F(y)\Vert - \Vert x-y\Vert \bigr | \le \xi \Vert x-y\Vert . \end{aligned}$$

We are interested in $\xi$-distortion maps with small $\xi$. (3) can be equivalently written as

$$\begin{aligned} (1-\xi )\Vert x-y\Vert \le \Vert F(x)-F(y)\Vert \le (1+\xi )\Vert x-y\Vert , \end{aligned}$$

and it is clear that when $\xi <1$, a $\xi$-distortion map is a bi-Lipschitz map. For our purposes the metric distortion constant $\xi$ is more convenient than a bi-Lipschitz constant. It is easy to show that if *F* is a $\xi$-distortion map, with $\xi <1$, then *F* is a homeomorphism onto its image, and $F^{-1}$ is a $({\xi }/({1-\xi }))$-distortion map (see Lemma A.1 (i)).

Assuming that $F_p|_{\varvec{\sigma }}$ is a $\xi$-distortion map for each *m*-simplex $\varvec{\sigma }\in {\mathscr {C}}_p$, we can bound how much it displaces points. Specifically, for any point $x \in |{\mathscr {C}}_p|$, we will bound $\Vert x-F(x)\Vert$. We exploit the fact that the $m+1$ vertices of $\varvec{\sigma }$ remain fixed, and use *trilateration*, i.e., we use the estimates of the distances to the fixed vertices to estimate the location of *F*(*x*). Here, the quality of the simplex comes into play.

#### Notation 2.9

(*simplex quality*)   If *p* is a vertex of $\varvec{\sigma }$, the *altitude* of *p* is the distance from *p* to the opposing facet of $\varvec{\sigma }$ and is denoted $a_p(\varvec{\sigma })$. The *thickness* of $\varvec{\sigma }$, denoted $t(\varvec{\sigma })$ (or just *t* if there is no risk of confusion), is given by *a*/(*mL*), where $a=a(\varvec{\sigma })$ is the smallest altitude of $\varvec{\sigma }$, and $L=L(\varvec{\sigma })$ is the length of the longest edge. We set $t(\varvec{\sigma })=1$ if $\varvec{\sigma }$ has dimension 0.

#### Lemma 2.10

(trilateration)   Suppose $\varvec{\sigma }\subset {\mathbb {R}}^m$ is an *m*-simplex, and $F:\varvec{\sigma }\rightarrow {\mathbb {R}}^m$ is a $\xi$-distortion map that leaves the vertices of $\varvec{\sigma }$ fixed. If $\xi \le 1$, then for any $x \in \varvec{\sigma }$,

$$\begin{aligned} \Vert x-F(x)\Vert \le \dfrac{3\xi L}{t}, \end{aligned}$$

where *L* is the length of the longest edge of $\varvec{\sigma }$, and *t* is its thickness.

#### Proof

Let $\{p_0,\ldots ,p_m\}$ be the vertices of $\varvec{\sigma }$. For $x \in \varvec{\sigma }$, let ${\tilde{x}} = F(x)$. We choose $p_0$ as the origin, and observe that

4 $$\begin{aligned} {p_i}^{\mathsf {T}}x = \dfrac{\Vert x\Vert ^2 + \Vert p_i\Vert ^2 -\Vert x-p_i\Vert ^2}{2}, \end{aligned}$$

which we write in matrix form as ${P}^{\mathsf {T}}x = b$, where *P* is the $m \times m$ matrix whose *i*-th column is $p_i$, and *b* is the vector whose *i*-th component is given by the right-hand side of (4). Similarly, we have ${P}^{\mathsf {T}}{\tilde{x}} = {\tilde{b}}$ with the obvious definition of ${\tilde{b}}$. Then

$$\begin{aligned} {\tilde{x}} - x = ({P}^{\mathsf {T}})^{-1}({\tilde{b}} - b). \end{aligned}$$

Since $F(p_0) = p_0 = 0$, we have $|{\Vert {\tilde{x}}\Vert - \Vert x\Vert }| \le \xi \Vert x\Vert$, and so

$$\begin{aligned} |{\Vert {\tilde{x}}\Vert ^2 - \Vert x\Vert ^2}|\le \xi (2 + \xi ) \Vert x\Vert ^2 \le 3 \xi L^2. \end{aligned}$$

Similarly, $|{\Vert x-p_i\Vert ^2-\Vert {\tilde{x}}-p_i\Vert ^2}|<3\xi L^2$. Thus $|{\smash {{\tilde{b}}_i}-b_i}|\le 3\xi L^2$, and $\Vert \smash {{\tilde{b}}}-b\Vert \le 3\sqrt{m}\,\xi L^2$. By [4, Lem. 2.4] we have $\Vert ({P}^{\mathsf {T}})^{-1}\Vert \le (\sqrt{m}\, t L)^{-1}$, and the stated bound follows. $\square$

#### Using ${\underline{\mathrm {St}}}(p)$ as ${\widetilde{{\mathscr {C}}}}_p$

For the local complex ${\widetilde{{\mathscr {C}}}}_p \subset {\mathscr {A}}$ introduced in Definition 2.4, we now make a specific choice: ${\widetilde{{\mathscr {C}}}}_p ={\underline{\mathrm {St}}}(p)$. This is the smallest complex allowed by the definition. For convenience, we define ${\hat{p}}={\widehat{\varPhi }}_p(p)$, so that ${\mathscr {C}}_p = {\widehat{\varPhi }}_p({\widetilde{{\mathscr {C}}}}_p) = {\underline{\mathrm {St}}}({\hat{p}})$.

**Fig. 1:**
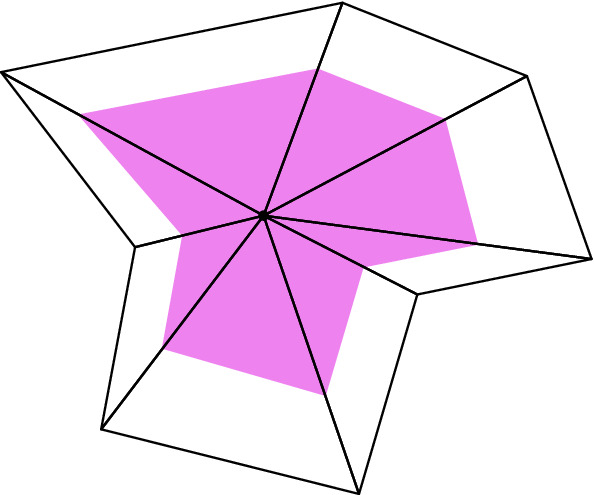
The open set $V_p$ (shaded) is a homothetically shrunk copy of the interior of ${\underline{\mathrm {St}}}({\hat{p}})$

We define $V_p$ to be the open set obtained by homothetically “shrinking” $|{\underline{\mathrm {St}}}({\hat{p}})|$ such that it is just large enough to contain the barycentres of the simplices that have ${\hat{p}}$ as a vertex (see Fig. 1). To be more specific we define $V_p$ to be the open set consisting of the points in $|{\underline{\mathrm {St}}}({\hat{p}})|$ whose barycentric coordinate with respect to ${\hat{p}}$ is strictly larger than $1/(m+1)-\delta$, where $\delta >0$ is arbitrarily small. Since the barycentric coordinates in each *m*-simplex sum to 1, and the piecewise linear maps ${\widehat{\varPhi }}_p$ preserve barycentric coordinates, this ensures that the sets ${\widehat{\varPhi }}_p^{-1}(V_p)$ cover $|{\mathscr {A}}|$.

We assume that $F_p$ is a $\xi$-distortion map on each simplex. The idea is to show that $F_p$ is an embedding on $V_p$. In order to employ the simplexwise positive embedding lemma (Lemma 2.7), we need to establish that there is a point in $V_p\setminus |{\mathscr {C}}_p^{m-1}|$ that is not mapped to the image of any other point in $|{\mathscr {C}}_p|$. We choose the barycentre of a simplex for this purpose. We say that a simplicial complex is a *pure* *m*-dimensional simplicial complex if every simplex is the face of an *m*-simplex.

##### Lemma 2.11

(a point covered once)   Suppose ${\mathscr {C}}$ is a pure *m*-dimensional finite simplicial complex embedded in ${\mathbb {R}}^m$, and that for each $\varvec{\sigma }\in {\mathscr {C}}$ we have $t(\varvec{\sigma }) \ge t_0$. Let $\varvec{\sigma }\in {\mathscr {C}}$ be an *m*-simplex with the largest diameter, i.e., $L(\varvec{\sigma }) \ge L(\varvec{\tau })$ for all $\varvec{\tau }\in {\mathscr {C}}$, and let *b* be the barycentre of $\varvec{\sigma }$. If $F :|{\mathscr {C}}| \rightarrow {\mathbb {R}}^m$ leaves the vertices of ${\mathscr {C}}$ fixed, and its restriction to any *m*-simplex in ${\mathscr {C}}$ is a $\xi$-distortion map with

5 $$\begin{aligned} \xi \le \dfrac{1}{6}\cdot \dfrac{mt_0^2}{m+1}, \end{aligned}$$

then $$F^{-1}(F(b)) = \{b\}$$.

##### Proof

Since $\xi < 1$, the restriction of *F* to $\varvec{\sigma }$ is injective. Suppose $x\in |{\mathscr {C}}|$ is such that $F(x)=F(b)$, but $x\ne b$. Then *x* belongs to some *m*-simplex $\varvec{\tau }\in {\mathscr {C}}$ different from $\varvec{\sigma }$. Since the distance from *b* to the boundary of $\varvec{\sigma }$ is

$$\begin{aligned} \dfrac{a(\varvec{\sigma })}{m+1} = \dfrac{mt(\varvec{\sigma })L(\varvec{\sigma })}{m+1}, \end{aligned}$$

it follows that $\Vert x-b\Vert >{mt(\varvec{\sigma })L(\varvec{\sigma })}/({m+1})$. But using Lemma 2.10 and the constraint (5) we arrive at a contradiction:

$$\begin{aligned} \Vert x-b\Vert \le \Vert {b-F(b)}\Vert + \Vert {x-F(x)}\Vert \le \dfrac{3\xi L(\varvec{\sigma })}{t(\varvec{\sigma })}+ \dfrac{3\xi L(\varvec{\tau })}{t(\varvec{\tau })}\le \dfrac{mt(\varvec{\sigma })L(\varvec{\sigma })}{m+1}. \end{aligned}$$

$$\square $$

Now we also need to ensure that $F_p(V_p) \cap F_p(|\partial {{\mathscr {C}}_p}|) = \emptyset$. Here we will explicitly use the assumption that ${\mathscr {C}}_p$ is ${\underline{\mathrm {St}}}({\hat{p}})$. We say that ${\underline{\mathrm {St}}}({\hat{p}})$ is a *full star* if its carrier is an *m*-manifold with boundary and ${\hat{p}}$ does not belong to $\partial {{\underline{\mathrm {St}}}({\hat{p}})}$.

##### Lemma 2.12

(barycentric boundary separation)   Suppose ${\underline{\mathrm {St}}}({\hat{p}})$ is a full *m*-dimensional star embedded in ${\mathbb {R}}^m$. Let $a_0 = \min _{\varvec{\sigma }\in {\underline{\mathrm {St}}}({\hat{p}})}a_{{\hat{p}}}(\varvec{\sigma })$ be the smallest altitude of ${\hat{p}}$ in the *m*-simplices in ${\underline{\mathrm {St}}}({\hat{p}})$. Suppose $x\in \varvec{\sigma }\in {\underline{\mathrm {St}}}({\hat{p}})$, where $\varvec{\sigma }$ is an *m*-simplex, and $\lambda _{\varvec{\sigma },{\hat{p}}}(x)$, the barycentric coordinate of *x* with respect to ${\hat{p}}$ in $\varvec{\sigma }$, satisfies $\lambda _{\varvec{\sigma },{\hat{p}}}(x) \ge \alpha$. Then $d_{{\mathbb {R}}^m}(x,|\partial {{\underline{\mathrm {St}}}({\hat{p}})}|) \ge \alpha a_0$. If $t_0$ is a lower bound on the thicknesses of the simplices in ${\underline{\mathrm {St}}}({\hat{p}})$, and $s_0$ is a lower bound on their diameters, then $d_{{\mathbb {R}}^m}(x,|\partial {{\underline{\mathrm {St}}}({\hat{p}})}|) \ge \alpha m t_0 s_0$.

##### Proof

Since we are interested in the distance to the boundary, consider a point $y \in |\partial {{\underline{\mathrm {St}}}({\hat{p}})}|$ such that the segment [*x*, *y*] lies in $|{\underline{\mathrm {St}}}({\hat{p}})|$. The segment passes through a sequence of *m*-simplices, $\varvec{\sigma }_0=\varvec{\sigma },\varvec{\sigma }_1, \dots , \varvec{\sigma }_n$, that partition it into subsegments $[x_i,y_i] \subset \varvec{\sigma }_i$ with $x_0=x$, $y_n=y$, and $x_i=y_{i-1}$ for all $i\in \{1,\dots ,n\}$.

Observe that $\lambda _{\varvec{\sigma }_i,{\hat{p}}}(x_i)=\lambda _{\varvec{\sigma }_{i-1},{\hat{p}}}(y_{i-1})$, and that

$$\begin{aligned} \Vert x_i-y_i\Vert \ge a_{{\hat{p}}}(\varvec{\sigma }_i)|{\lambda _{\varvec{\sigma }_i,{\hat{p}}}(x_i)-\lambda _{\varvec{\sigma }_i,{\hat{p}}}(y_i)}|. \end{aligned}$$

Thus

$$\begin{aligned} \begin{aligned} |x-y|&= \sum _{i=0}^n |x_i-y_i|\ge \sum _{i=0}^n a_{{\hat{p}}}(\varvec{\sigma }_i)|{\lambda _{\varvec{\sigma }_i,{\hat{p}}}(x_i)-\lambda _{\varvec{\sigma }_i,{\hat{p}}}(y_i)}|\\&\ge a_0\sum _{i=0}^n(\lambda _{\varvec{\sigma }_i,{\hat{p}}}(x_i)-\lambda _{\varvec{\sigma }_i,{\hat{p}}}(y_i))= a_0 (\lambda _{\varvec{\sigma },{\hat{p}}}(x)-\lambda _{\varvec{\sigma }_n,{\hat{p}}}(y)) \\&= a_0\lambda _{\varvec{\sigma },{\hat{p}}}(x)\ge a_0 \alpha . \end{aligned} \end{aligned}$$

From the definition of thickness we find that $a_0\ge t_0 m s_0$, yielding the second statement of the lemma. $\square$

Lemma 2.12 allows us to quantify the distortion bound that we need to ensure that the boundary of ${\underline{\mathrm {St}}}({\hat{p}})$ does not get mapped by $F_p$ into the image of the open set $V_p$. The argument is the same as for Lemma 2.11, but there we were only concerned with the barycentre of the largest simplex, so the relative sizes of the simplices were not relevant as they are here (compare the bounds (5) and (6)).

##### Lemma 2.13

(boundary separation for $V_p$)   Suppose ${\underline{\mathrm {St}}}({\hat{p}})$ is a full star embedded in ${\mathbb {R}}^m$, and every *m*-simplex $\varvec{\sigma }$ in ${\underline{\mathrm {St}}}({\hat{p}})$ satisfies $s_0 \le L(\varvec{\sigma }) \le L_0$, and $t(\varvec{\sigma })\ge t_0$. If the restriction of $F_p$ to any *m*-simplex in ${\underline{\mathrm {St}}}({\hat{p}})$ is a $\xi$-distortion map, with

6 $$\begin{aligned} \xi < \dfrac{1}{6}\cdot \dfrac{m}{m+1}\cdot \dfrac{s_0t_0^2}{L_0}, \end{aligned}$$

then $F_p(V_p) \cap F_p(|\partial {{\underline{\mathrm {St}}}({\hat{p}})}|) = \emptyset$, where $V_p$ is the set of points with barycentric coordinate with respect to ${\hat{p}}$ in a containing *m*-simplex strictly greater than $1/(m+1)-\delta$, with $\delta >0$ an arbitrary, sufficiently small parameter.

##### Proof

If $x \in \varvec{\sigma }\in {\underline{\mathrm {St}}}({\hat{p}})$ has barycentric coordinate with respect to ${\hat{p}}$ larger than ${1}/({m+1})-\delta$, and $y \in \varvec{\tau }\in \partial {{\underline{\mathrm {St}}}({\hat{p}})}$, then Lemmas 2.10 and 2.12 ensure that $F_p(x) \ne F_p(y)$ provided

$$\begin{aligned} \dfrac{3\xi L(\varvec{\sigma })}{t(\varvec{\sigma })}+\dfrac{3\xi L(\varvec{\tau })}{t(\varvec{\tau })}\le \biggl (\dfrac{1}{m+1}-\delta \biggr )m s_0t_0, \end{aligned}$$

which is satisfied by (6) when $\delta >0$ satisfies

$$\begin{aligned} \delta \le \dfrac{1}{m+1} - \dfrac{6L_0\xi }{m s_0 t_0^2}. \end{aligned}$$

$$\square $$

When inequality (6) (and therefore also (5)) is satisfied, we can employ the embedding lemma (Lemma 2.7) to guarantee that $V_p$ is embedded:

##### Lemma 2.14

(local homeomorphism)  Suppose ${\mathscr {A}}$ is a compact *m*-manifold complex (without boundary), with vertex set ${\mathscr {P}}$, and $M$ is an *m*-manifold. A map $H :|{\mathscr {A}}| \rightarrow M$ is a covering map if the following criteria are satisfied:

(i) **compatible atlases** There are compatible atlases for *H*, with ${\widetilde{{\mathscr {C}}}}_p = {\underline{\mathrm {St}}}(p)$ for each $p \in {\mathscr {P}}$ (Definition 2.4).

For the charts in the compatible atlases we have the following:

(ii) **simplex quality** For each $p \in {\mathscr {P}}$, every simplex $\varvec{\sigma }\in {\underline{\mathrm {St}}}({\hat{p}})= {\widehat{\varPhi }}_p({\underline{\mathrm {St}}}(p))$ satisfies $s_0 \le L(\varvec{\sigma }) \le L_0$ and $t(\varvec{\sigma })\ge t_0$ (Notation 2.9).
(iii) **distortion control** For each $p\in {\mathscr {P}}$, the map
  $$\begin{aligned} F_p = \phi _p \circ H \circ {\widehat{\varPhi }}_p^{-1}:|{\underline{\mathrm {St}}}({\hat{p}})| \rightarrow {\mathbb {R}}^m, \end{aligned}$$
  when restricted to any *m*-simplex in ${\underline{\mathrm {St}}}({\hat{p}})$, is an orientation-preserving $\xi$-distortion map with
  $$\begin{aligned} \xi < \dfrac{m s_0 t_0^2}{6(m+1)L_0} \end{aligned}$$
  (Definitions B.5 and 2.8).

### Injectivity

Having established that *H* is a covering map, to ensure that *H* is injective it suffices to demonstrate that on each component of $M$ there is a point with only a single point in its preimage. Injectivity follows since the number of points in the preimage is locally constant for covering maps.

Since each simplex is embedded by *H*, it is sufficient to show that for each vertex $q \in {\mathscr {P}}$, if $H(q) \in H(\varvec{\sigma })$, then *q* is a vertex of $\varvec{\sigma }$. This ensures that $H^{-1}(H(q)) = \{q\}$, and by Remark 2.5 each component of $M$ must contain the image of a vertex.

In practice, we typically don’t obtain this condition directly. The complex ${\mathscr {A}}$ is *constructed* by means of the local patches ${\mathscr {C}}_p$, and it is with respect to these patches that the vertices behave well.

#### Definition 2.15

(*vertex sanity*)  If $H :|{\mathscr {A}}| \rightarrow M$ has compatible atlases (Definition 2.4), then *H* exibits *vertex sanity* if: for all vertices $p,q \in {\mathscr {P}}$, if $\phi _p \circ H(q) \in |{\underline{\mathrm {St}}}({\hat{p}})| ={\widehat{\varPhi }}_p(|{\underline{\mathrm {St}}}(p)|)$, then *q* is a vertex of ${\underline{\mathrm {St}}}(p)$.

Together with the distortion bounds that are imposed on $F_p$, Definition 2.15 ensures that the image of a vertex cannot lie in the image of a simplex to which it does not belong:

#### Lemma 2.16

(injectivity)   If $H :|{\mathscr {A}}| \rightarrow M$ satisfies the hypotheses of Lemma 2.14 as well as Definition 2.15, then *H* is injective, and therefore a homeomorphism.

#### Proof

Towards a contradiction, suppose that $H(q) \in H(\varvec{\sigma })$ and that *q* is not a vertex of the *m*-simplex $\varvec{\sigma }$. This means there is some $x \in \varvec{\sigma }$ such that $H(x) = H(q)$. Let *p* be a vertex of $\varvec{\sigma }$. The vertex sanity hypothesis (Definition 2.15) implies that $\phi _p \circ H(q)$ must be either outside of $|{\underline{\mathrm {St}}}({\hat{p}})|$, or belong to its boundary. Thus Lemmas 2.12 and 2.10, and the bound on $\xi$ from Lemma 2.14 (iii) imply that the barycentric coordinate of *x* with respect to *p* must be smaller than $\tfrac{1}{/}({m+1})$: Let ${\hat{x}}= {\widehat{\varPhi }}_p(x)$, and ${\hat{\varvec{\sigma }}}={\widehat{\varPhi }}_p(\varvec{\sigma })$. Lemma 2.10 says that

$$\begin{aligned} |{F_p({\hat{x}})-{\hat{x}}}| \le \dfrac{3\xi L_0}{t_0} < \dfrac{m s_0 t_0}{2(m+1)}\le \dfrac{a_0}{2(m+1)}, \end{aligned}$$

where $a_0$ is a lower bound on the altitudes of ${\hat{p}}$, as in Lemma 2.12. Since $F_p({\hat{x}})=\phi _p \circ H(x)$ is at least as far away from ${\hat{x}}$ as $\partial {{\underline{\mathrm {St}}}({\hat{p}})}$, Lemma 2.12 implies that the barycentric coordinate of ${\hat{x}}\in {\hat{\varvec{\sigma }}}$ with respect to ${\hat{p}}$ must be no larger than $1/(2(m+1))$. Since ${\widehat{\varPhi }}_p$ preserves barycentric coordinates, and the argument works for any vertex *p* of $\varvec{\sigma }$, we conclude that all the barycentric coordinates of *x* in $\varvec{\sigma }$ are strictly less than $1/(m+1)$. We have reached a contradiction with the fact that the barycentric coordinates of *x* must sum to 1. $\square$

### Main Result

To recap, Lemmas 2.14 and 2.16 yield the following triangulation result. In the bound on $\xi$ from Lemma 2.14 (iii), we replace the factor ${m}/(m+1)$ with 1/2, the lower bound attained when $m=1$.

#### Theorem 2.17

(triangulation)   Suppose ${\mathscr {A}}$ is a compact *m*-manifold complex (without boundary), with vertex set ${\mathscr {P}}$, and $M$ is an *m*-manifold. A map $H :|{\mathscr {A}}| \rightarrow M$ is a homeomorphism if the following criteria are satisfied:

(i) **compatible atlases** There are compatible atlases
  $$\begin{aligned} \{({\widetilde{{\mathscr {C}}}}_p,{\widehat{\varPhi }}_p)\}_{p\in {\mathscr {P}}}, \quad {\widetilde{{\mathscr {C}}}}_p \subset {\mathscr {A}},\quad and \quad \{(U_p,\phi _p)\}_{p\in {\mathscr {P}}}, \quad U_p \subset M, \end{aligned}$$
  for *H*, with ${\widetilde{{\mathscr {C}}}}_p = {\underline{\mathrm {St}}}(p)$ for each $p \in {\mathscr {P}}$, the vertex set of ${\mathscr {A}}$ (Definition 2.4).

For the charts in the compatible atlases we have the following:

(ii) **simplex quality** For each $p \in {\mathscr {P}}$, every simplex $\varvec{\sigma }\in {\underline{\mathrm {St}}}({\hat{p}})= {\widehat{\varPhi }}_p({\underline{\mathrm {St}}}(p))$ satisfies $s_0 \le L(\varvec{\sigma }) \le L_0$ and $t(\varvec{\sigma }) \ge t_0$ (Notation 2.9).
(iii) **distortion control**   For each $p\in {\mathscr {P}}$, the map
  $$\begin{aligned} F_p = \phi _p \circ H \circ {\widehat{\varPhi }}_p^{-1}:|{\underline{\mathrm {St}}}({\hat{p}})| \rightarrow {\mathbb {R}}^m, \end{aligned}$$
  when restricted to any *m*-simplex in ${\underline{\mathrm {St}}}({\hat{p}})$, is an orientation-preserving $\xi$-distortion map with
  $$\begin{aligned} \xi < \dfrac{s_0 t_0^2}{12L_0} \end{aligned}$$
  (Definitions B.5 and 2.8).
(iv) **vertex sanity** For all vertices $p,q\in {\mathscr {P}}$, if $\phi _p\circ H(q)\in |{\underline{\mathrm {St}}}({\hat{p}})|$, then *q* is a vertex of ${\underline{\mathrm {St}}}(p)$.

Heuristically speaking the first criterion ensures, among others, that all the stars of vertices completely lie in a coordinate neighbourhood. Without this condition there could be parts that are not covered by the simplicial complex. The criteria (ii) and (iii) prevent the orientation of a simplex to change from one map to another, while the vertex sanity condition prevents multiple covers.

#### Remark 2.18

The constants $L_0$, $s_0$, and $t_0$ that constrain the simplices in the local complex ${\underline{\mathrm {St}}}({\hat{p}})$, and the metric distortion of $F_p$ in Theorem 2.17 can be considered to be local, i.e., they may depend on $p\in {\mathscr {P}}$.

## Submanifolds of Euclidean Space

As a specific application of Theorem 2.17, we consider a smooth (or at least $C^2$) compact *m*-dimensional submanifold of Euclidean space: $M\subset {\mathbb {R}}^N$. A simplicial complex ${\mathscr {A}}$ is built whose vertices are a finite set ${\mathscr {P}}$ sampled from the manifold: ${\mathscr {P}}\subset M$. The motivating model for this setting is the tangential Delaunay complex [8]. In that case ${\mathscr {A}}$ is constructed as a subcomplex of a weighted Delaunay triangulation of ${\mathscr {P}}$ in the ambient space ${\mathbb {R}}^N$, so it is necessarily embedded. However, in the general setting discussed in this paper we do not need to assume *a priori* that ${\mathscr {A}}$ is embedded in ${\mathbb {R}}^N$. (This does not force us to consider ${\mathscr {A}}$ to be abstract in the combinatorial sense. In particular, the simplices are Euclidean simplices, not just sets of vertices.) Instead, we assume only that the embedding of the vertex set ${\mathscr {P}}\hookrightarrow {\mathbb {R}}^N$ defines an *immersion* $\iota :|{\mathscr {A}}| \rightarrow {\mathbb {R}}^N$. By this we mean that for any vertex $p\in {\mathscr {P}}$ we have that the restriction of $\iota$ to $|{\underline{\mathrm {St}}}(p)|$ is an embedding.

At each point $x\in M$, the tangent space $T_xM\subset T_x{\mathbb {R}}^N$ is naturally viewed as an *m*-dimensional affine flat in ${\mathbb {R}}^N$, with the vector-space structure defined by taking the distinguished point *x* as the origin. The maps involved in Theorem 2.17 will be defined by projection maps. The coordinate charts are defined using the orthogonal projection $\mathrm {pr}_{\!T_pM}:{\mathbb {R}}^N \rightarrow T_pM$. As discussed in Sect. 3.3, for a sufficiently small neighbourhood $U_p \subset M$, we obtain an embedding

$$\begin{aligned} \phi _p = \mathrm {pr}_{\!T_pM}|_{U_p}:U_p \subset M\rightarrow T_pM\cong {\mathbb {R}}^m, \end{aligned}$$

which will define our coordinate maps for $M$.

For the map $H:|{\mathscr {A}}| \rightarrow M$, we will employ the closest point projection map defined in Sect. 3.1 and discussed further in Sect. 3.4. There is an open neighbourhood $U_{\!M}\subset {\mathbb {R}}^N$ of $M$ on which each point has a unique closest point on $M$, so the closest-point projection map $\mathrm {pr}_{\!M}:U_{\!M}\rightarrow M$ is well defined. We define $H = \mathrm {pr}_{\!M}\circ \iota$.

As demanded by Definition 2.4, for each $p\in {\mathscr {P}}$ the coordinate map ${\widehat{\varPhi }}_p$ for ${\mathscr {A}}$ is the secant map of $\phi _p \circ H$ restricted to ${\widetilde{{\mathscr {C}}}}_p=|{\underline{\mathrm {St}}}(p)|$, and since $\mathrm {pr}_{\!T_pM}$ is already a linear map, and $\mathrm {pr}_{\!M}$ is the identity on the vertices, this means ${\widehat{\varPhi }}_p = \mathrm {pr}_{\!T_pM}\circ \iota |_{|{\underline{\mathrm {St}}}(p)|}$.

In Sects. 3.1 and 3.2 we review some of the geometric concepts and standard results that we will use in the rest of the section. In order to bound the metric distortion of the maps $F_p = \phi _p \circ H\circ {\widehat{\varPhi }}_p^{-1}$ via Lemma A.1, we are free to choose any convenient metric on $M$. We employ here the metric of the ambient space ${\mathbb {R}}^N$, rather than the intrinsic metric of geodesic distances.

### The Reach

Since $M\subset {\mathbb {R}}^N$ is compact, for any $x \in {\mathbb {R}}^N$ there is a point $z \in M$ that realizes the distance to $M$, i.e.,

$$\begin{aligned} \delta _{M}(x) :=d_{{\mathbb {R}}^N}(x,M):= \inf _{y\in M}d_{{\mathbb {R}}^N}(x,y) = d_{{\mathbb {R}}^N}(x,z). \end{aligned}$$

The *medial axis* of $M$ is the set of points $\mathop {\mathrm {ax}}\nolimits (M) \subset {\mathbb {R}}^N$ that have more than one such closest point on $M$. In other words, if $x\in \mathop {\mathrm {ax}}\nolimits (M)$, then an open ball $B_{{\mathbb {R}}^N}(x,r)$, centred at *x* and of radius $r=\delta _{M}(x)$, will be tangent to $M$ at two or more distinct points. The *cut locus* of $M$ is the closure of the medial axis, and is denoted ${\overline{\mathop {\mathrm {ax}}\nolimits }}(M)$. The *reach* of $M$ is defined by $\mathop {\mathrm {rch}}\nolimits (M):=d_{{\mathbb {R}}^N}(M,{\overline{\mathop {\mathrm {ax}}\nolimits }}(M))$. We observe below that for compact $C^2$ submanifolds, $\mathop {\mathrm {rch}}\nolimits (M)>0$. Thus, by definition, every point *x* in the open neighbourhood $U_{\!M}:= {\mathbb {R}}^N\setminus {\overline{\mathop {\mathrm {ax}}\nolimits }}(M)$ of $M$ has a unique closest point ${\check{x}}\in M$. The *closest-point projection* map

$$\begin{aligned} \mathrm {pr}_{\!M}:U_{\!M}\rightarrow M, \end{aligned}$$

takes *x* to this closest point: $\mathrm {pr}_{\!M}(x)={\check{x}}$. To each point $x\in M$, we associate a *normal space*

$$\begin{aligned} N_xM= \{n\in T_x{\mathbb {R}}^N\mid n\varvec{\cdot }v= 0\,\;{\mathrm{for}~\mathrm{all}}\,v\in T_xM\} \end{aligned}$$

of vectors orthogonal to $T_xM$. Thus $T_x{\mathbb {R}}^N= N_xM\oplus T_xM$. As with the tangent space, the normal space at *x* is naturally identified with an affine flat in ${\mathbb {R}}^N$. It has dimension $k=N-m$ and is orthogonal to $T_xM$.

#### Definition 3.1

The reach of $M$ is the distance between the medial axis and $M$ itself.

#### Remark 3.2

Most of the results below can be localized in terms of the local feature size [1, 19] (or similar quantities), see [7] for details.

### Affine Flats and Angles

The angle between two vectors $u,v \in {\mathbb {R}}^N \setminus \{0\}$ is denoted $\angle (u,v)$ (this angle is $\le \pi$). If $K \subseteq {\mathbb {R}}^n$ is a linear subspace, $\mathrm {pr}_{\!K}(u)$ is the orthogonal projection of *u* into *K*. We define $\angle (u,K)$ to be $\pi /2$ if $\mathrm {pr}_{\!K}(u) = 0$, and otherwise $\angle (u,K) = \angle (u,\mathrm {pr}_{\!K}(u))$ (thus $\angle (u,K)\le \pi /2$). If *K* and *L* are two linear subspaces, then

$$\begin{aligned} \angle (K,L) = \sup _{u \in K \setminus \{0\}}\angle (u,L). \end{aligned}$$

The definition is only interesting when $\dim K \le \dim L$. Observe that if $\dim K = \dim L$, then $\angle (K,L)=\angle (L,K)$. If *K* and *L* are affine flats in ${\mathbb {R}}^N$, then $\angle (K,L)$ is the angle between the corresponding parallel vector subspaces. If $\varvec{\sigma }$ is a simplex with $\dim \varvec{\sigma }\le \dim L$, then $\angle (\varvec{\sigma },L):=\angle (\mathrm {aff}(\varvec{\sigma }),L)$, where $\mathrm {aff}(\varvec{\sigma })$ is the affine hull of $\varvec{\sigma }$. We denote the orthogonal complement of a linear subspace $K \subseteq {\mathbb {R}}^N$ by $K^{\perp }$. A short exercise yields the following observations:

#### Lemma 3.3

 

(i) If *K*, *L* are subspaces of ${\mathbb {R}}^N$, then $\angle (L^\perp ,K^\perp ) = \angle (K,L)$.
(ii) If $Q \subset {\mathbb {R}}^N$ is a subspace of codimension 1, then $\angle (Q^\perp ,K)= \pi /2 - \angle (K,Q)$.

We now recall a number of results starting with [19, Thm. 4.8 (7)].

#### Lemma 3.4

Given any two points $x,y \in M\subset {\mathbb {R}}^N$, we have

(i) $$\displaystyle \sin \angle ([x,y],T_xM) \le \dfrac{|x-y|}{2\mathop {\mathrm {rch}}\nolimits (M)}$$,
(ii) $$\displaystyle d_{{\mathbb {R}}^N}(y,T_xM) \le \dfrac{|x-y|^2}{2\mathop {\mathrm {rch}}\nolimits (M)}$$.

We will also use the following result, Lemma 6 and Corollary 3 of [10]:

#### Lemma 3.5

(tangent space variation)   Let $x,y \in M$. Then

$$\begin{aligned} \sin \angle (T_xM,T_yM)\le \dfrac{|y-x|}{\mathop {\mathrm {rch}}\nolimits (M)}\quad \text {and}\quad \angle (T_xM,T_yM)\le \dfrac{\pi |y-x|}{2\mathop {\mathrm {rch}}\nolimits (M)}. \end{aligned}$$

#### Remark 3.6

In the proof of the lemma above the fact that $M$ is $C^2$ is used, however alternatives with worse constants exist for general manifolds with positive reach [10]. In particular all results in this section hold for general manifolds of positive reach albeit with worse constants.

We will need to bound the angle between a simplex with vertices on $M$ and the nearby tangent spaces. To this end we employ a result established by Whitney [24, p. 127] in the formulation presented in [4, Lem. 2.1]:

#### Lemma 3.7

(Whitney’s angle bound)  Suppose $\varvec{\sigma }$ is a *j*-simplex whose vertices all lie within a distance $\eta$ from a *k*-dimensional affine space, $K\subset {\mathbb {R}}^N$, with $k\ge j$. Then

$$\begin{aligned} \sin \angle (\varvec{\sigma },K) \le \dfrac{2\eta }{tL}, \end{aligned}$$

where *t* is the thickness of $\varvec{\sigma }$ and *L* is the length of its longest edge.

The following lemma can be found in [3, Lem. 7.9].

#### Lemma 3.8

(simplices lie close to $M$)   Let $\varvec{\sigma }$ be a simplex with vertices on $M$. Then for all $x,y \in \varvec{\sigma }$,

$$\begin{aligned} d_{{\mathbb {R}}^N}(y,T_{{\check{x}}}M)< \dfrac{2L(\varvec{\sigma })^2}{\mathop {\mathrm {rch}}\nolimits (M)},\quad \text {and in particular,} \quad \delta _{M}(x) < \dfrac{2L(\varvec{\sigma })^2}{\mathop {\mathrm {rch}}\nolimits (M)}, \end{aligned}$$

where $${\check{x}}= \mathrm {pr}_{\!M}(x)$$.

#### Lemma 3.9

(simplex-tangent space angle bounds) Suppose $\varvec{\sigma }\subset {\mathbb {R}}^N$ is a simplex of dimension $\le m$ with vertices on $M$. If *p* is a vertex of $\varvec{\sigma }$, then

(i) if *p* is a vertex of $\varvec{\sigma }$, $\displaystyle \sin \angle (\varvec{\sigma },T_pM) \le \dfrac{L}{t\mathop {\mathrm {rch}}\nolimits (M)}$;
(ii) if $x\in \varvec{\sigma }$ and ${\check{x}}= \mathrm {pr}_{\!M}(x)$, then $\displaystyle \sin \angle (\varvec{\sigma },T_{{\check{x}}}M)\le \dfrac{3L}{t\mathop {\mathrm {rch}}\nolimits (M)}$.

#### Proof

(i) By Lemma 3.4 (ii), all the vertices of $\varvec{\sigma }$ are within a distance $\eta =L^2/(2\mathop {\mathrm {rch}}\nolimits (M))$ from $T_pM$, and so Lemma 3.7 ensures

$$\begin{aligned} \sin \angle (\varvec{\sigma },T_pM) \le \dfrac{2\eta }{tL} =\dfrac{L}{t\mathop {\mathrm {rch}}\nolimits (M)}. \end{aligned}$$

(ii) Because $|x-{\check{x}}|< L(\varvec{\sigma })$, and $|x-p|< L(\varvec{\sigma })$, we see that $|p-{\check{x}}|< 2L(\varvec{\sigma })$. Lemma 3.5 now yields

$$\begin{aligned} \sin \angle (T_pM,T_{{\check{x}}}M) \le \dfrac{|p-{\check{x}}|}{\mathop {\mathrm {rch}}\nolimits (M)}\le \dfrac{2L}{\mathop {\mathrm {rch}}\nolimits (M)}, \end{aligned}$$

and the result follows using part (i). $\square$

### Distortion of Orthogonal Projection: ${\widehat{\varPhi }}_p$ and $\phi _p$

The coordinate maps $\phi _p$ and ${\widehat{\varPhi }}_p$ are defined in terms of the orthogonal projection to $T_pM$. The size of the neighbourhoods used to define the coordinate charts are constrained by the requirement that these maps be embeddings, which we establish by ensuring that they are $\xi$-distortion maps with $\xi <1$. The proof of the following lemma can be found in [3, Lem. 7.14].

#### Lemma 3.10

(definition and distortion of $\phi _p$)   Let $U_p = B_{{\mathbb {R}}^N}(p,r) \cap M$, where $r=\rho \mathop {\mathrm {rch}}\nolimits (M)$, with $\rho <1/2$. Define $\phi _p :=\mathrm {pr}_{\!T_pM}|_{U_p}$. Then $\phi _p$ is a $\xi$-distortion map with $\xi = 4\rho ^2$.

#### Remark 3.11

(differential of $\phi _p$)   It is straightforward to verify from the definitions that for any $x\in U_p$,

$$\begin{aligned} d(\mathrm {pr}_{\!T_pM}|_{M})_x = \mathrm {pr}_{\!T_pM}|_{T_xM}. \end{aligned}$$

The domain of the map ${\widehat{\varPhi }}_p$, i.e., an upper bound on the allowable size of the simplices in $\iota (|{\underline{\mathrm {St}}}(p)|)$, is governed by the following bound on the metric distortion of the projection from a simplex. Here $d(f)_{x}$ denotes the differential of the function *f* at the point *x*.

#### Lemma 3.12

(simplexwise distortion of ${\widehat{\varPhi }}_p$)   Suppose $\varvec{\sigma }\subset {\mathbb {R}}^N$ is a simplex of dimension $\le m$ with vertices on $M$. If *p* is a vertex of $\varvec{\sigma }$, and

$$\begin{aligned} L(\varvec{\sigma }) < t(\varvec{\sigma })\mathop {\mathrm {rch}}\nolimits (M), \end{aligned}$$

then the restriction of $\mathrm {pr}_{\!T_pM}$ to $\varvec{\sigma }$ is a $\xi$-distortion map with

$$\begin{aligned} \xi = \biggl (\dfrac{L(\varvec{\sigma })}{t(\varvec{\sigma })\mathop {\mathrm {rch}}\nolimits (M)}\biggr )^{\!2}. \end{aligned}$$

#### Proof

Let $x,y \in \varvec{\sigma }$ and set ${\hat{x}}= \mathrm {pr}_{\!T_pM}(x)$, ${\hat{y}}= \mathrm {pr}_{\!T_pM}(y)$. By Lemma 3.9 (i),

$$\begin{aligned} \sin \angle (\varvec{\sigma },T_pM) \le \dfrac{L}{t\mathop {\mathrm {rch}}\nolimits (M)}. \end{aligned}$$

So, putting $\theta =\angle ([x,y],[{\hat{x}},{\hat{y}}]) \le \angle (\varvec{\sigma },T_pM)$ we find

$$\begin{aligned} \begin{aligned} |x-y| - |{\hat{x}}-{\hat{y}}|&= (1 - \cos \theta )|x-y|\\&\le (1 - \cos ^2 \theta )|x-y|\le \biggl (\dfrac{L}{t\mathop {\mathrm {rch}}\nolimits (M)}\biggr )^{\!2}|x-y|. \end{aligned} \end{aligned}$$

The result follows since $|{\hat{x}}-{\hat{y}}|\le |x-y|$. $\square$

Therefore, in order to use our framework, we need to ensure that for each simplex $\varvec{\sigma }\in {\mathscr {A}}$, the simplex $\iota (\varvec{\sigma }) \subset {\mathbb {R}}^N$ satisfies $L < t \mathop {\mathrm {rch}}\nolimits (M)$. Then, in conformance with Sect. 2.1.1, we set ${\widehat{\varPhi }}_p = \mathrm {pr}_{\!T_pM} \circ \iota |_{|{\underline{\mathrm {St}}}(p)|}$, and we *require* that it be an embedding. Although we have established that $\mathrm {pr}_{\!T_pM}$ is an embedding on each simplex, and we have assumed that $\iota |_{|{\underline{\mathrm {St}}}(p)|}$ is an embedding, these criteria do not imply that ${\widehat{\varPhi }}_p$ is an embedding. We leave this as a requirement of the embedding theorem (requirement (a) of Theorem 3.16), i.e., something that needs to be established in context. For the case of the tangential Delaunay complex, the embedding follows naturally because $\phi _p(|{\underline{\mathrm {St}}}(p)|)$ is seen as a weighted Delaunay triangulation in $T_pM$ [8].

### Distortion of the Closest-Point Projection Map: *H*

Recall that $H:|{\mathscr {A}}| \rightarrow M$ is the map that we wish to show is a homeomorphism. In our current context *H* is based on the closest-point projection map $\mathrm {pr}_{\!M}:{\mathbb {R}}^N \rightarrow M$. As discussed at the beginning of this section, we define $H = \mathrm {pr}_{\!M}\circ \iota$, where $\iota :|{\mathscr {A}}|\rightarrow {\mathbb {R}}^N$ is the immersion of our simplicial complex into ${\mathbb {R}}^N$. Once *H* is shown to be a homeomorphism, it follows that $\iota$ is in fact an embedding, but we don’t assume this a priori. The metric on $|{\mathscr {A}}|$ (i.e., the edge lengths of the Euclidean simplices) is *defined* by $\iota$, so $\iota$ itself does not contribute to the metric distortion of *H*.

We will be needing the following result from [19, Thm. 4.8 (8)]:

#### Lemma 3.13

(upper bound for $\mathrm {pr}_{\!M}$ distortion)   Let $x,y \in U_{\!M}$ and ${\check{x}}=\mathrm {pr}_{\!M}(x)$, and ${\check{y}}=\mathrm {pr}_{\!M}(y)$, as usual. If $a \ge \max {\{\delta _{M}(x),\delta _{M}(y)\}}$ for some $a<\mathop {\mathrm {rch}}\nolimits (M)$, then

7 $$\begin{aligned} |{\check{y}}-{\check{x}}| \le \biggl (1-\frac{a}{\mathop {\mathrm {rch}}\nolimits (M)} \biggr )^{\!-1}|y-x|. \end{aligned}$$

#### Lemma 3.14

(simplexwise distortion of *H*)   Suppose $\varvec{\sigma }\subset U_{\!M}$ is a simplex of dimension $\le m$ whose vertices lie in $M$. If $L(\varvec{\sigma }) < t(\varvec{\sigma })\mathop {\mathrm {rch}}\nolimits (M)/3$, then the restriction of $\mathrm {pr}_{\!M}$ to $\varvec{\sigma }$ is a $\xi$-distortion map with

$$\begin{aligned} \xi = \dfrac{12L^2}{t^2\mathop {\mathrm {rch}}\nolimits (M)^2}. \end{aligned}$$

#### Proof

By Lemma 3.8,

8 $$\begin{aligned} \delta _{M}(x) < a= \dfrac{2L^2}{\mathop {\mathrm {rch}}\nolimits (M)}\ \quad \text {for any } \ x\in \varvec{\sigma }. \end{aligned}$$

Thus it follows from Lemma 3.13 that

9 $$\begin{aligned} |{\check{y}}-{\check{x}}| \le \biggl (1-\dfrac{2L^2}{\mathop {\mathrm {rch}}\nolimits (M)^2}\biggr )^{\!-1}|y-x|\le \biggl (1+ \dfrac{4L^2}{\mathop {\mathrm {rch}}\nolimits (M)^2}\biggr )|y-x|, \end{aligned}$$

for any $$x,y\in \varvec{\sigma }$$.

We now need to establish a lower bound on $|{\check{y}}-{\check{x}}|$. Let $Q_x$ be the hyperplane through ${\check{x}}$ and orthogonal to $[x,{\check{x}}]$, and let ${\widehat{y}}$ and ${\widehat{{\check{y}}}}$ be the orthogonal projection of *y* and ${\check{y}}$ into $Q_x$. We have

10 $$\begin{aligned} |{\check{y}}-{\check{x}}| \ge \bigl |{{\widehat{{\check{y}}}}-{\check{x}}}\bigr | \ge |{\widehat{y}}-{\check{x}}| - \bigl |{{\widehat{{\check{y}}}}-{\widehat{y}}}\bigr |. \end{aligned}$$

To get a lower bound on $|{\widehat{y}}-{\check{x}}| =|y-x|\cos \angle ([x,y],Q_x)$, notice that

$$\begin{aligned} \angle ([x,y],Q_x) \le \angle (\mathrm {aff}(\varvec{\sigma }),Q_x)\le \angle (\mathrm {aff}(\varvec{\sigma }),T_{{\check{x}}}M), \end{aligned}$$

and by Lemma 3.9(ii),

$$\begin{aligned} \sin \angle (\mathrm {aff}(\varvec{\sigma }),T_{{\check{x}}}M) \le \dfrac{3L}{t\mathop {\mathrm {rch}}\nolimits (M)}. \end{aligned}$$

Thus,

$$\begin{aligned} \cos \angle ([x,y],Q_x)\ge \biggl (1 - \biggl (\dfrac{3L}{t\mathop {\mathrm {rch}}\nolimits (M)}\biggr )^{\!2}\biggr )^{\!1/2}\ge 1 - \dfrac{9L^2}{t^2\mathop {\mathrm {rch}}\nolimits (M)^2}, \end{aligned}$$

and so

11 $$\begin{aligned} |{\widehat{y}}-{\check{x}}| \ge \biggl (1 - \dfrac{9L^2}{t^2\mathop {\mathrm {rch}}\nolimits (M)^2}\biggr )|y-x|. \end{aligned}$$

To get an upper bound on the second term on the right side of (10), let $Q_y$ be the hyperplane through ${\check{y}}$ and orthogonal to $[{\check{y}},y]$. We have

$$\begin{aligned} \begin{aligned} |{{\widehat{{\check{y}}}}-{\widehat{y}}}|&= |{\check{y}}-y|\cos \angle ([{\check{y}},y],Q_x)\\&= |{\check{y}}-y|\sin \angle (Q_x,Q_y)\quad \text {by Lemma }3.3\,\mathrm{(ii)}\\&\le |{\check{y}}-y|\sin \angle (T_{{\check{x}}},T_{{\check{y}}})\\&\le |{\check{y}}-y|\dfrac{|{\check{y}}-{\check{x}}|}{\mathop {\mathrm {rch}}\nolimits (M)}\quad \quad \quad \qquad \text {by Lemma }3.5\\&\le \dfrac{2L^2}{\mathop {\mathrm {rch}}\nolimits (M)^2}|{\check{y}}-{\check{x}}|\quad \quad \quad \quad \, \text {by }(8). \end{aligned} \end{aligned}$$

Now, using (9), we have

12 $$\begin{aligned} |{{\widehat{{\check{y}}}}-{\widehat{y}}}|\le \dfrac{2L^2}{\mathop {\mathrm {rch}}\nolimits (M)^2}\biggl (1+\dfrac{4L^2}{\mathop {\mathrm {rch}}\nolimits (M)^2}\biggr )|y-x|< \dfrac{3L^2}{\mathop {\mathrm {rch}}\nolimits (M)^2} |y-x|, \end{aligned}$$

since the hypothesis $3L/{\mathop {\mathrm {rch}}\nolimits (M)} < t$ implies $4L^2/{\mathop {\mathrm {rch}}\nolimits (M)^2}<1/2$. Finally, plugging (11) and (12) back into (10), we get

$$\begin{aligned} |{\check{y}}-{\check{x}}| \ge \biggl (1-\dfrac{12L^2}{t^2\mathop {\mathrm {rch}}\nolimits (M)^2} \biggr )|y-x|. \end{aligned}$$

Comparing this lower bound with (9), we arrive at the stated value for the metric distortion $\xi$. $\square$

### Triangulation Criteria for Submanifolds

In order to employ Theorem 2.17 we first ensure that we meet the compatible atlases criteria (Definition 2.4). In Lemma 3.10 we defined

$$\begin{aligned} U_p = B_{{\mathbb {R}}^N}(p,r) \cap M,\ \ \quad \text {where }\ r = \rho \mathop {\mathrm {rch}}\nolimits (M)< \dfrac{\mathop {\mathrm {rch}}\nolimits (M)}{2}. \end{aligned}$$

We need to ensure that $H(|{\underline{\mathrm {St}}}(p)|) \subseteq U_p$. In our context, this means that we require $\mathrm {pr}_{\!M}(\iota (|{\underline{\mathrm {St}}}(p)|))\subset U_p$, and Lemma 3.8 ensures that if $L(\varvec{\sigma })\le L_0$ for all $\varvec{\sigma }\in \iota ({\underline{\mathrm {St}}}(p))$, then it is sufficient to choose $r=L_0+ 2L_0^2{/}\mathop {\mathrm {rch}}\nolimits (M)$, or

13 $$\begin{aligned} \rho = \dfrac{L_0}{\mathop {\mathrm {rch}}\nolimits (M)}\biggl (1+\dfrac{2L_0}{\mathop {\mathrm {rch}}\nolimits (M)}\biggr ). \end{aligned}$$

Our bound on $L_0$ itself will be much smaller than $\mathop {\mathrm {rch}}\nolimits (M)$.

We need to establish the metric distortion of $F_p = \phi _p \circ H \circ {\widehat{\varPhi }}_p^{-1}$ restricted to any *m*-simplex in ${\widehat{\varPhi }}_p(\iota ({\underline{\mathrm {St}}}(p)))$, and ensure that it meets the distortion control criterion of Theorem 2.17. Anticipating the bound we will need to meet the distortion-control criterion of Theorem 2.17, we impose the constraint

14 $$\begin{aligned} \dfrac{L_0^2}{t_0^2\mathop {\mathrm {rch}}\nolimits (M)^2}\le \dfrac{1}{16^2}, \end{aligned}$$

where $t_0$ is a lower bound on the thickness: $t(\varvec{\sigma })\ge t_0$ for all $\varvec{\sigma }\in \iota ({\underline{\mathrm {St}}}(p))$. We remark that $L_0$ and $\mathop {\mathrm {rch}}\nolimits (M)$ may be considered to be *local constants*, i.e., they may depend on the vertex $p \in {\mathscr {P}}$, however $t_0$ and the ratio $L_0{/}\mathop {\mathrm {rch}}\nolimits (M)$ will be global constants.

Using (14) together with Lemmas 3.12 and A.1 (i), we can bound the metric distortion of ${\widehat{\varPhi }}_p^{-1}$ as

$$\begin{aligned} \xi _1 = \dfrac{L^2}{t^2\mathop {\mathrm {rch}}\nolimits (M)^2}\biggl (1-\dfrac{L^2}{t^2\mathop {\mathrm {rch}}\nolimits (M)^2}\biggr )^{\!-1}\le \dfrac{16^2}{16^2-1}\cdot \dfrac{L_0^2}{t_0^2\mathop {\mathrm {rch}}\nolimits (M)^2}. \end{aligned}$$

Lemma 3.14 gives us the distortion of *H*:

$$\begin{aligned} \xi _2 = 12\dfrac{L_0^2}{t_0^2\mathop {\mathrm {rch}}\nolimits (M)^2}. \end{aligned}$$

For $\phi _p$, using Lemma 3.10 and (13) we get the distortion bound

$$\begin{aligned} \xi _3 = 4\rho ^2= \dfrac{4L_0^2}{\mathop {\mathrm {rch}}\nolimits (M)^2}\biggl (1 + \dfrac{2L_0}{\mathop {\mathrm {rch}}\nolimits (M)} \biggr )^{\!2}\le \dfrac{9}{2}\cdot \dfrac{L_0^2}{t_0^2\mathop {\mathrm {rch}}\nolimits (M)^2}. \end{aligned}$$

Lemma A.1 (ii) says that the distortion of $F_p$ is no more than

$$\begin{aligned} \xi = \xi _1 + \xi _2 + \xi _3 + \xi _1\xi _2 + \xi _1\xi _3 + \xi _2\xi _3+ \xi _1\xi _2\xi _3. \end{aligned}$$

Using (14) we find that $F_p$ is a $\xi$-distortion map with

15 $$\begin{aligned} \xi = \frac{19L_0^2}{t_0^2\mathop {\mathrm {rch}}\nolimits (M)^2}. \end{aligned}$$

Observe that (14) implies that all the maps involved have distortion less than 1.

Now we need to ensure that this bound meets the distortion-bound requirement for Theorem 2.17. We have chosen to use here the properties of the Euclidean simplices in the ambient space ${\mathbb {R}}^N$, but in Theorem 2.17 we are considering simplices in the local coordinate space; for us these are the projected simplices, e.g., ${\hat{\varvec{\sigma }}}={\widehat{\varPhi }}_p(\varvec{\sigma })=\mathrm {pr}_{\!T_pM}(\varvec{\sigma })$. Using Lemma 3.12, the distortion properties of the affine map $\mathrm {pr}_{\!T_pM}$ imply

$$\begin{aligned} a({\hat{\varvec{\sigma }}}) \ge \biggl (1 - \dfrac{L_0^2}{t_0^2\mathop {\mathrm {rch}}\nolimits (M)^2}\biggr )a(\varvec{\sigma }),\qquad L({\hat{\varvec{\sigma }}}) \le L(\varvec{\sigma }), \end{aligned}$$

and therefore

$$\begin{aligned} t({\hat{\varvec{\sigma }}}) \ge \biggl (1 - \dfrac{L_0^2}{t_0^2\mathop {\mathrm {rch}}\nolimits (M)^2}\biggr )t(\varvec{\sigma }). \end{aligned}$$

We can thus set

$$\begin{aligned} {\hat{t}}_0= \biggl (1 - \dfrac{L_0^2}{t_0^2\mathop {\mathrm {rch}}\nolimits (M)^2}\biggr )t_0,\quad {\hat{L}}_0= L_0,\quad {\hat{s}}_0= \biggl (1 - \dfrac{L_0^2}{t_0^2\mathop {\mathrm {rch}}\nolimits (M)^2}\biggr )s_0, \end{aligned}$$

where $s_0$ is a lower bound for the diameters of the simplices in $\iota ({\underline{\mathrm {St}}}(p))$. Then, in order to meet the distortion control criterion of Theorem 2.17, we require

16 $$\begin{aligned} \frac{19L_0^2}{t_0^2\mathop {\mathrm {rch}}\nolimits (M)^2} < \frac{{\hat{s}}_0{\hat{t}}_0^2}{12{\hat{L}}_0}= \biggl (1 - \dfrac{L_0^2}{t_0^2\mathop {\mathrm {rch}}\nolimits (M)^2}\biggr )^{\!3}\frac{s_0t_0^2}{12L_0}. \end{aligned}$$

It is convenient to define $\mu _0= s_0/L_0$. Then, observing that

$$\begin{aligned} \biggl (1 - \dfrac{L_0^2}{t_0^2\mathop {\mathrm {rch}}\nolimits (M)^2}\biggr )^{\!3} \ge 1 - 3\dfrac{L_0^2}{t_0^2\mathop {\mathrm {rch}}\nolimits (M)^2}, \end{aligned}$$

we see that (16) is satisfied if

17 $$\begin{aligned} L_0^2 \le \frac{\mu _0t_0^4\mathop {\mathrm {rch}}\nolimits (M)^2}{16^2}. \end{aligned}$$

Our constraint (17) on $L_0$ induces a bound on $\rho$, as defined in (13),

18 $$\begin{aligned} \rho \mathop {\mathrm {rch}}\nolimits (M)\le \frac{9}{8}L_0\le \frac{9}{2^7}\mathop {\mathrm {rch}}\nolimits (M). \end{aligned}$$

We now only need to establish that $F_p$ is simplexwise positive to arrive at our triangulation theorem for submanifolds.

#### Lemma 3.15

$F_p$ is simplexwise positive on $\mathrm {pr}_{\!T_pM}(\iota (|{\underline{\mathrm {St}}}(p)|))$.

#### Proof

Recall that $F_p = \phi _p \circ H \circ {\widehat{\varPhi }}_p^{-1}$, and observe that the restriction of $F_p$ to an *m*-simplex ${{\hat{\varvec{\sigma }}}}\in \mathrm {pr}_{\!T_pM}(\iota ({\underline{\mathrm {St}}}(p)))$ is differentiable. The bound (17) together with (15) implies $F_p$ is a $\xi$-distortion map with $\xi <1$, so Lemma A.2 ensures that for any *m*-simplex $\varvec{\sigma }\in \mathrm {pr}_{\!T_pM}(\iota ({\underline{\mathrm {St}}}(p)))$, the differential of $F_p$ does not vanish on $\varvec{\sigma }$.

We choose an orientation on $U_p$; thus we have an orientation on each tangent space $T_xM$, $x\in U_p$. The projection map $\mathrm {pr}_{\!T_pM}|_{T_xM} =d(\mathrm {pr}_{\!T_pM}|_{M})_x = d(\phi _p)_x$ (see Remark 3.11) is then orientation preserving, because of continuity: it is certainly true when $x=p$, and Lemmas 3.10 and A.2 imply that the differential $d(\phi _p)$ is nondegenerate on $U_p$. Thus $\phi _p$ is simplexwise positive.

For an *m*-simplex $\varvec{\sigma }\in \iota ({\underline{\mathrm {St}}}(p))$ we *define* the orientation such that $\mathrm {pr}_{\!T_pM}|_{\varvec{\sigma }}$ is positive. Equivalently, we define the orientation on $\iota (|{\underline{\mathrm {St}}}(p)|)$ to be such that ${\widehat{\varPhi }}_p$ is simplexwise positive. This is not problematic, since we require that ${\widehat{\varPhi }}_p$ be an embedding. Thus ${\widehat{\varPhi }}_p$ is simplexwise positive by our definitions.

It remains to show that $\mathrm {pr}_{\!M}|_{\varvec{\sigma }}$ is positive for each *m*-simplex $\varvec{\sigma }\in \iota ({\underline{\mathrm {St}}}(p))$. First observe that for any $x\in {\mathbb {R}}^N$, the kernel of $d(\mathrm {pr}_{\!M})_x$ is the subspace of $T_x{\mathbb {R}}^N$ corresponding to $N_{{\check{x}}}M$ (under the canonical identification $T_x{\mathbb {R}}^N \cong {\mathbb {R}}^N \cong T_{{\check{x}}}{\mathbb {R}}^N$, where ${\check{x}}=\mathrm {pr}_{\!M}(x)$). Also, observe that if $x\in M$, then $d(\mathrm {pr}_{\!M}|_{T_xM})_x = \mathop {\mathrm {id}}\nolimits _{T_xM}$. This is easily seen by noting that $d(\mathrm {pr}_{\!M}|_{T_xM})_x = d(\mathrm {pr}_{\!M})_x|_{T_xM}$ and using curves on $M$ to apply the definition of the differential.

Thus at the central vertex $p \in \varvec{\sigma }$, we can express $d(H|_{\varvec{\sigma }})_p$ as

$$\begin{aligned} d(\mathrm {pr}_{\!M}|_{\varvec{\sigma }})_p = \mathop {\mathrm {id}}\nolimits _{T_pM} \circ \mathrm {pr}_{\!T_pM}|_{\varvec{\sigma }}. \end{aligned}$$

Since we have established that $\mathrm {pr}_{\!T_pM}|_{\varvec{\sigma }}$ is positive, it follows that $d(\mathrm {pr}_{\!M}|_{\varvec{\sigma }})_p$ is positive, and since $d(\mathrm {pr}_{\!M}|_{\varvec{\sigma }})$ is nondegenerate on $\varvec{\sigma }$ (because $d(F_p)$ is), it follows that $H|_{\iota (|{\underline{\mathrm {St}}}(p)|)}$ is simplexwise positive. Thus $F_p$ is orientation preserving on each simplex of ${\underline{\mathrm {St}}}({\hat{p}})$. $\square$

#### Theorem 3.16

(triangulation for submanifolds)   Let $M\subset {\mathbb {R}}^N$ be a compact $C^2$ manifold, and ${\mathscr {P}}\subset M$ a finite set of points such that for each connected component $M_{c}$ of $M$, $M_{c}\cap {\mathscr {P}}\ne \emptyset$. Suppose that ${\mathscr {A}}$ is a simplicial complex whose vertices, ${\mathscr {A}}^0$, are identified with ${\mathscr {P}}$, by a bijection ${\mathscr {A}}^0\rightarrow {\mathscr {P}}$ such that the resulting piecewise linear map $\iota :|{\mathscr {A}}| \rightarrow {\mathbb {R}}^N$ is an immersion, i.e., $\iota |_{|{\underline{\mathrm {St}}}(p)|}$ is an embedding for each vertex *p*. Assume that:

- For each vertex $p\in {\mathscr {P}}$, the projection $\mathrm {pr}_{\!T_pM}|_{\iota (|{\underline{\mathrm {St}}}(p)|)}$ is an embedding and *p* lies in the interior of $\mathrm {pr}_{\!T_pM}(\iota (|{\underline{\mathrm {St}}}(p)|))$.
- There are constants $0<t_0\le 1$, $0<\mu _0\le 1$, and $\varepsilon _0>0$ such that for each simplex $\varvec{\sigma }\in \iota ({\mathscr {A}})$, and each vertex $p \in \varvec{\sigma }$,
  $$\begin{aligned} t(\varvec{\sigma })\ge t_0,\quad \mu _0\varepsilon _0\mathop {\mathrm {rch}}\nolimits (M)\le L(\varvec{\sigma }) \le \varepsilon _0\mathop {\mathrm {rch}}\nolimits (M),\quad \varepsilon _0 \le \dfrac{\mu _0^{1/2}t_0^2}{16}. \end{aligned}$$
- For any vertices $p,q \in {\mathscr {P}}$, if $q \in U_p = B_{{\mathbb {R}}^N}(p,r)\cap M$, where $r=\mathop {\mathrm {rch}}\nolimits (M)/14$, then $\mathrm {pr}_{\!T_pM}(q) \in \mathrm {pr}_{\!T_pM}(\iota ({\underline{\mathrm {St}}}(p)))$ if and only if *q* is a vertex of ${\underline{\mathrm {St}}}(p)$.

Then:

(i) $\iota$ is an embedding, so the complex ${\mathscr {A}}$ may be identified with $\iota ({\mathscr {A}})$.
(ii) The closest-point projection map $\mathrm {pr}_{\!M}|_{|{\mathscr {A}}|}$ is a homeomorphism $|{\mathscr {A}}| \rightarrow M$.
(iii) For any $x \in \varvec{\sigma }\in {\mathscr {A}}$,
  $$\begin{aligned} \delta _{M}(x) = |{\check{x}}-x| \le 2\varepsilon _0^2\mathop {\mathrm {rch}}\nolimits (M),\quad \sin \angle (\varvec{\sigma }, T_{{\check{x}}}) \le \dfrac{3\varepsilon _0}{t_0}, \end{aligned}$$
  where $${\check{x}}= \mathrm {pr}_{\!M}(x)$$.

#### Proof

Observe that the local embedding condition (a) implies that ${\mathscr {A}}$ is a compact *m*-manifold without boundary. Condition (b) is a reformulation of (17). Condition (c) is the vertex sanity condition of Theorem 2.17, we only have to verify that we have chosen our neighbourhood large enough. Indeed using (18) we see $r=9\mathop {\mathrm {rch}}\nolimits (M)/128 < \mathop {\mathrm {rch}}\nolimits (M)/14$.

Thus, from our argument above, the criteria of Theorem 2.17 are satisfied, and $H = \mathrm {pr}_{\!M}\circ \iota$ is a homeomorphism. It follows that $\iota$ is injective, and since $|{\mathscr {A}}|$ is compact, $\iota$ must be an embedding. The second consequence, that $\mathrm {pr}_{\!M}|_{|{\mathscr {A}}|}$ is a homeomorphism, is now immediate. For the third consequence using Lemma 3.8 and condition (b) gives

$$\begin{aligned} \delta _{M}(x) \le \dfrac{2L^2}{\mathop {\mathrm {rch}}\nolimits (M)}\le \dfrac{2\varepsilon _0^2 \mathop {\mathrm {rch}}\nolimits (M)^2}{\mathop {\mathrm {rch}}\nolimits (M)}\le 2 \varepsilon _0^2 \mathop {\mathrm {rch}}\nolimits (M). \end{aligned}$$

The second inequality follows from Lemma 3.9 (ii). $\square$

## Strong Differential Bounds and Riemannian Manifolds

In this section we will fit the triangulation criteria for Riemannian manifolds in the general framework provided by Theorem 2.17. To do so we first develop triangulation criteria for maps where we have control on the distortion thanks to bounds on the differential of the map $F_p$, see point (iv) of Proposition 4.5 for details. We believe that this abstract view of the triangulation criteria may be of independent interest.

The triangulation criteria of Theorem 2.17 presented in Sect. 2 have been inspired by the criteria in the Riemannian setting [16, Prop. 16], so the many intermediate results can be used without modification. However one of the conditions in [16, Prop. 16] was too weak and injectivity was in fact not assured. The need to close this gap further motivates this section.

### Strong Differential Bounds

It is not difficult to derive bounds on the metric distorion, assuming one has bounds on the differential of a map. More precisely, for $F_p$ to be a $\xi$-distortion map it is sufficient to show that for any vector *w* tangent to a point *u* in the domain of $F_p$,

19 $$\begin{aligned} (1-\xi )|w| \le |{d(F_p)_u w}| \le (1+\xi )|w|, \end{aligned}$$

see Lemma A.3. Following [16, Prop. 16] we are however interested in a stronger bound on the differential. We require that

20 $$\begin{aligned} \Vert {d(F_p)_u -\mathop {\mathrm {id}}\nolimits }\Vert \le \xi \end{aligned}$$

for all *u* in the domain of $F_p$ (with $p \in {\mathscr {P}}$ fixed). We stress that the bound (20) is strictly stronger than (19). It is not difficult to establish that (20) implies that $F_p$ is a $\xi$-distortion map [16, Lem. 11] on each simplex. However, whereas (19) only constrains how much $dF_p$ can change the magnitude of a vector, (20) also constrains how much the direction can change. For this kind of bound on the differential, there is no need to exploit the trilateration lemma (Lemma 2.10):

#### Lemma 4.1

[16, Lem. 12] Suppose $\varvec{\omega }\subseteq {\mathbb {R}}^m$ is a convex set and $F:\varvec{\omega }\rightarrow {\mathbb {R}}^m$ is a smooth map with a fixed point $p \in \varvec{\omega }$. If

$$\begin{aligned} \Vert dF_x - {{\,\mathrm{Id}\,}}\Vert \le \xi \ \quad \text {for all }\ x \in \varvec{\omega }, \end{aligned}$$

then

$$\begin{aligned} |{F(x)-x}| \le \xi |{x-p}|\ \quad \text {for all }\ x \in \varvec{\omega }. \end{aligned}$$

The added control obtained by the strong bound (20) on the differential enables us to ensure that $F_p$ embeds the boundary of ${\underline{\mathrm {St}}}(p)$. This in turn implies that $F_p$ embeds all of ${\underline{\mathrm {St}}}(p)$. This follows from the following corollary to Whitney’s lemma [24, Lemma AII.15a] (which is demonstrated as Lemmas B.9 and 2.7 in this work). We include the proof here since the proof in [16] erroneously states that Whitney’s proof implies that a simplexwise positive map is a local homeomorphism; this is not true, but such a map is an open map.

#### Definition 4.2

(*smooth on* ${\mathscr {C}}$)  Given a simplicial complex ${\mathscr {C}}$, we say that a map $F:|{\mathscr {C}}| \rightarrow {\mathbb {R}}^m$ is *smooth on* ${\mathscr {C}}$ if for each $\varvec{\sigma }\in {\mathscr {C}}$ the restriction $F |_{\varvec{\sigma }}$ is smooth.

#### Lemma 4.3

[16, Lem. 13] Let ${\mathscr {C}}$ be a (finite) simplicial complex embedded in ${\mathbb {R}}^m$ such that ${{\,\mathrm{int}\,}}(|{\mathscr {C}}|)$ is nonempty and connected, and $\partial {|{\mathscr {C}}|}$ is a compact, connected $(m{-}1)$-manifold. Suppose $F :|{\mathscr {C}}| \rightarrow {\mathbb {R}}^m$ is smooth on ${\mathscr {C}}$ and simplexwise positive. If the restriction of *F* to $\partial {|{\mathscr {C}}|}$ is an embedding, then *F* is a topological embedding.

#### Proof

The assumptions on ${{\,\mathrm{int}\,}}(|{\mathscr {C}}|)$ and $\partial {|{\mathscr {C}}|}$ imply that ${\mathscr {C}}$ is a pure *m*-complex, and that each $(m{-}1)$-simplex is either a boundary simplex, or the face of exactly two *m*-simplices. (This is a nontrivial exercise, and requires the demand that $\partial {|{\mathscr {C}}|}$ be connected, which was absent in the original statement of the lemma.)

By the same argument as in the proof of Lemma 2.7, $F({{\,\mathrm{int}\,}}(|{\mathscr {C}}|))$ is open. By the Jordan–Brouwer separation theorem [22, Sect. IV.7], ${\mathbb {R}}^n \setminus F(\partial {|{\mathscr {C}}|})$ consists of two open components, one of which is bounded. Since $F(|{\mathscr {C}}|)$ is compact, Lemma B.9 implies that $F({{\,\mathrm{int}\,}}(|{\mathscr {C}}|))$ must coincide with the bounded component, and in particular $F({{\,\mathrm{int}\,}}(|{\mathscr {C}}|)) \cap F(\partial {|{\mathscr {C}}|})=\emptyset$, so $F({{\,\mathrm{int}\,}}(|{\mathscr {C}}|))$ is a single connected component.

We need to show that *F* is injective. First we observe that the set of points in $F({{\,\mathrm{int}\,}}(|{\mathscr {C}}|))$ that have exactly one point in the preimage is nonempty. It suffices to look in a neigbhourhood of a point $y \in F(\partial {|{\mathscr {C}}|})$. Choose $y = F(x)$, where *x* is in the relative interior of $\varvec{\sigma }^{m-1} \subset \partial {|{\mathscr {C}}|}$. Then there is a neighbourhood *V* of *y* such that *V* does not intersect the image of any other simplex of dimension less than or equal to $n-1$. Let $\varvec{\sigma }^m$ be the unique *m*-simplex that has $\sigma ^{m-1}$ as a face. Then $F^{-1}(V \cap F(|{\mathscr {C}}|) \subset \varvec{\sigma }^m$, and it follows that every point in $V \cap {{\,\mathrm{int}\,}}(|{\mathscr {C}}|)$ has a unique point in its image. Now the injectivity of *F* follows from Lemma 2.7. $\square$

We then obtain bounds on the differential of $F_p$ sufficient to ensure that it embeds ${\underline{\mathrm {St}}}(p)$:

#### Lemma 4.4

[16, Lem. 14] Suppose ${\mathscr {C}}= {\underline{\mathrm {St}}}({\hat{p}})$ is a $t_0$-thick, pure *m*-complex embedded in ${\mathbb {R}}^m$ such that all of the *m*-simplices are incident to a single vertex, ${\hat{p}}$, and ${\hat{p}}\in {{\,\mathrm{int}\,}}(|{\mathscr {C}}|)$ (i.e., ${\underline{\mathrm {St}}}({\hat{p}})$ is a full star). If $F :|{\mathscr {C}}| \rightarrow {\mathbb {R}}^m$ is smooth on ${\mathscr {C}}$, and satisfies

21 $$\begin{aligned} \Vert dF - {{\,\mathrm{Id}\,}}\Vert < mt_0\end{aligned}$$

on each *m*-simplex of ${\mathscr {C}}$, then *F* is an embedding.

If the requirements of Lemma 4.4 are met, then *H* is a local homemorphism, and we are left with ensuring that it is injective, in order to guarantee that it is a homeomorphism. To this end we employ the vertex sanity assumption (Definition 2.15), and we arrive at the following triangulation result, which can replace the flawed [16, Prop. 16]:

#### Proposition 4.5

(triangulation with differential control)   Suppose ${\mathscr {A}}$ is a compact *m*-manifold complex (without boundary), with vertex set ${\mathscr {P}}$, and $M$ is an *m*-manifold. A map $H :|{\mathscr {A}}| \rightarrow M$ is a homeomorphism if the following criteria are satisfied:

(i) **compatible atlases** There are compatible atlases
  $$\begin{aligned} \{({\widetilde{{\mathscr {C}}}}_p,{\widehat{\varPhi }}_p)\}_{p\in {\mathscr {P}}}, \quad {\widetilde{{\mathscr {C}}}}_p \subset {\mathscr {A}},\quad \ \text {and}\ \quad \{(U_p,\phi _p)\}_{p\in {\mathscr {P}}}, \quad U_p \subset M, \end{aligned}$$
  for *H*, with ${\widetilde{{\mathscr {C}}}}_p = {\underline{\mathrm {St}}}(p)$ for each $p \in {\mathscr {P}}$, the vertex set of ${\mathscr {A}}$ (Definition 2.4).

or the charts in the compatible atlases we have the following:

(ii) **simplex quality** For each $p \in {\mathscr {P}}$, every simplex $\varvec{\sigma }\in {\underline{\mathrm {St}}}({\hat{p}})= {\widehat{\varPhi }}_p({\underline{\mathrm {St}}}(p))$ satisfies $s_0 \le L(\varvec{\sigma }) \le L_0$ and $t(\varvec{\sigma }) \ge t_0$ (Notation 2.9).
(iii) **distortion control** For each $p\in {\mathscr {P}}$, the map
  $$\begin{aligned} F_p = \phi _p \circ H \circ {\widehat{\varPhi }}_p^{-1}:|{\underline{\mathrm {St}}}({\hat{p}})| \rightarrow {\mathbb {R}}^m, \end{aligned}$$
  is smooth on ${\underline{\mathrm {St}}}({\hat{p}})$, and simplexwise positive, and for any *m*-simplex $\varvec{\sigma }\in {\underline{\mathrm {St}}}({\hat{p}})$ and any $u\in \varvec{\sigma }$, we have
  $$\begin{aligned} \Vert {d(F_p)_u - \mathop {\mathrm {id}}\nolimits }\Vert < \xi = \dfrac{s_0 t_0}{2 L_0} \end{aligned}$$
  (Definitions 4.2 and B.8).
(iv) **vertex sanity** For all vertices $p,q \in {\mathscr {P}}$, if $\phi _p \circ H(q) \in |{\underline{\mathrm {St}}}({\hat{p}})|$, then *q* is a vertex of ${\underline{\mathrm {St}}}(p)$.

#### Proof

Since the requirements of Lemma 4.4 are met, we only need to show that *H* is injective. The argument the same as for Lemma 2.16, with minor modifications.

Towards a contradiction, suppose that $H(q) \in H(\varvec{\sigma })$ and that *q* is not a vertex of the *m*-simplex $\varvec{\sigma }$. This means there is some $x \in \varvec{\sigma }$ such that $H(x) = H(q)$. Let *p* be a vertex of $\varvec{\sigma }$. The vertex sanity hypothesis (Definition 2.15) implies that $\phi _p \circ H(q)$ must be either outside of $|{\underline{\mathrm {St}}}({\hat{p}})|$, or belong to its boundary. Thus Lemmas 2.12 and 4.1, and the bound on $\xi$ imply that the barycentric coordinate of *x* with respect to *p* must be smaller than $1/(m+1)$: Let ${\hat{x}}= {\widehat{\varPhi }}_p(x)$, and ${\hat{\varvec{\sigma }}}={\widehat{\varPhi }}_p(\varvec{\sigma })$. Lemma 4.1 says that

$$\begin{aligned} |{F_p({\hat{x}})-{\hat{x}}}| < \xi L_0 = \dfrac{s_0 t_0}{2} \le \dfrac{a_0}{2m}, \end{aligned}$$

where $a_0$ is a lower bound on the altitudes of ${\hat{p}}$, as in Lemma 2.12. Since $F_p({\hat{x}})=\phi _p \circ H(x)=\phi _p\circ H(q)$ is at least as far away from ${\hat{x}}$ as $\partial {{\underline{\mathrm {St}}}({\hat{p}})}$, Lemma 2.12 implies that the barycentric coordinate of ${\hat{x}}\in {\hat{\varvec{\sigma }}}$ with respect to ${\hat{p}}$ must be strictly less than ${1}/({2m})\le {1}/({m+1})$. Since ${\widehat{\varPhi }}_p$ preserves barycentric coordinates, and the argument works for any vertex *p* of $\varvec{\sigma }$, we conclude that all the barycentric coordinates of *x* in $\varvec{\sigma }$ are strictly less than ${1}/({m+1})$. We have reached a contradiction with the fact that the barycentric coordinates of *x* must sum to 1. $\square$

#### Remark 4.6

Notice that the constraint on $\xi$ in Proposition 4.5 is only linear in $t_0$, whereas it is quadratic in $t_0$ in Theorem 2.17.

### Triangulations of Riemannian Manifolds

We now apply the theory developed in the previous subsection to Riemannian simplices and triangulations, as we did in [16]. Because of the close relationship with [16] we will employ here the notation and conventions of that paper, which differ slightly from those in the rest of the current document. In particular, the dimension of the Riemannian manifold we are triangulating is *n*, and $\sigma$ denotes an abstract simplex, i.e., a set of vertices, usually on the manifold, $M$. When these vertices are lifted via the inverse of the exponential map to $T_pM$, the resulting vertex set is denoted $\sigma (p)$. A “filled in” Euclidean simplex is denoted $\varvec{\sigma }_{{\mathbb {E}}}$. The vertex set of ${\mathscr {A}}$ is denoted by $S$ instead of ${{\mathscr {P}}}$.

As we mentioned in the introduction of this section an extra assumption needs to be added to some statements of [16]. If the following hypothesis is added to Proposition 16 and Theorem 2, all results of [16] hold:

#### Hypothesis 4.7

(*simple injectivity assumption*)   If *q* is a vertex of ${\mathscr {A}}$ and $H(q)\in H(\varvec{\sigma }_{{\mathbb {E}}})$, then *q* is a vertex of $\varvec{\sigma }$.

The injectivity of the map *H* follows trivially from Hypothesis 4.7, since it has been established that *H* is a covering map. However, this assumption is not easy to verify, at least not in some applications of interest, e.g., [5]. We provide here corrected statements of the affected results, obtained by replacing [16, Prop. 16] with Proposition 4.5, which uses the vertex sanity assumption, Definition 2.15. Unfortunately, the bound required on the differential in Proposition 4.5 (iv) is qualitatively different from that imposed in [16, Prop. 16]. In particular, we need to impose a lower bound $s_0$ on the diameters of the simplices. It is convenient to define $\mu _0 = s_0/L_0$.

In the proof of [16, Thm. 2], Proposition 16 is employed at the bottom of page 23, just before the statement of the theorem. Reworking that short calculation, and incorporating the vertex sanity hypothesis, we obtain:

#### Theorem 4.8

([16, Thm. 2] corrected)   Suppose $M$ is a compact *n*-dimensional Riemannian manifold with sectional curvatures $K$ bounded by $|{K}| \le \varLambda$, and ${\mathscr {A}}$ is an abstract simplicial complex with finite vertex set $S\subset M$. Define quality parameters $t_0> 0$, $0< \mu _0 \le 1$, and let

22 $$\begin{aligned} h= \min {\biggl \{ \frac{\iota _{M}}{4},\frac{\sqrt{\mu _0}t_0}{6 \sqrt{\varLambda }} \biggr \}}. \end{aligned}$$

Suppose:

(i) For every $p \in S$, the vertices of ${\underline{\mathrm {St}}}(p)$ are contained in $B_{M}(p,h)$, and the balls $\{B_{M}(p,h)\}_{p \in S}$ cover $M$.
(ii) For every $p \in S$, the restriction of the inverse of the exponential map $\exp _p^{-1}$ to the vertices of ${\underline{\mathrm {St}}}(p)\subset {\mathscr {A}}$ defines a piecewise linear embedding of $|{\underline{\mathrm {St}}}(p)|$ into $T_{p}{M}$, realising ${\underline{\mathrm {St}}}(p)$ as a full star, $\widehat{{\underline{\mathrm {St}}}(p)}$, such that every simplex $\sigma (p)$ has thickness $t(\sigma (p)) \ge t_0$ and diameter $\mu _0L_0 \le L(\sigma (p)) \le L_0$.
(iii) For all vertices $p,q \in S$, if $(\exp _p|_{B_{M}(p,h)})^{-1}(q) \in |\widehat{{\underline{\mathrm {St}}}(p)}|$, then *q* is a vertex of ${\underline{\mathrm {St}}}(p)$.

Then ${\mathscr {A}}$ triangulates $M$, and the triangulation is given by the barycentric coordinate map on each simplex.

For [16, Prop. 26], the affected argument is in the paragraph preceding the statement of the proposition. New (stronger) bounds on *h* ensuring a piecewise flat metric on $\mathscr {A}$ are given when we replace that paragraph with (where it is understood that references to Theorem 2 are to the corrected version, Theorem 4.8):

Thus in order to guarantee that the $\ell _{ij}$ describe a non-degenerate Euclidean simplex, we require that $\varLambda h^2 =\eta t_0^2/2$, for some non-negative $\eta < 1$.

Under the conditions of Theorem 2 we may have $h^2 \varLambda ={\mu _0 t_0^2}/{36}$, which gives us $\eta ={\mu _0}/{18} \le {1}/{18} < 1$. Thus the requirements of Theorem 2 are sufficient to ensure the existence of a piecewise flat metric on ${\mathscr {A}}$, and we obtain:

#### Proposition 4.9

([16, Prop. 26] corrected)   If the requirements of Theorem 2 are satisfied, then the geodesic distances between the endpoints of the edges in ${\mathscr {A}}$ define a piecewise flat metric on ${\mathscr {A}}$ such that each simplex $\sigma \in {\mathscr {A}}$ satisfies

$$\begin{aligned} t(\sigma ) > \dfrac{3t_0}{4\sqrt{n}}. \end{aligned}$$

Likewise, for [16, Thm. 3], it is sufficient to impose the new bounds introduced in Theorem 4.8 to obtain the same metric distortion bound:

#### Theorem 4.10

([16, Thm. 3] corrected)   If the requirements of Theorem 2 are satisfied, then ${\mathscr {A}}$ is naturally equipped with a piecewise flat metric $d_{{\mathscr {A}}}$ defined by assigning to each edge the geodesic distance in $M$ between its endpoints. With this metric on ${\mathscr {A}}$, if $H :|{\mathscr {A}}| \rightarrow M$ is the triangulation defined by the barycentric coordinate map, then the metric distortion induced by *H* is quantified as

$$\begin{aligned} |{ d_{M}(H(x),H(y)) - d_{{\mathscr {A}}}(x,y) }| \le \dfrac{50\varLambda h^2}{t_0^2}d_{{\mathscr {A}}}(x,y), \end{aligned}$$

for all $$x,y \in |{\mathscr {A}}|$$.

Finally, we remark that [5, Thm. 3] employed [16, Thm. 3], but although some of the discussion leading up to the statement of the result should be modified to account for the new bound imposed by Theorem 4.10, the actual result [5, Thm. 3] stands as stated, since the bound on the sampling density required there is manifestly sufficient to accommodate (22), and the construction via local Delaunay triangulations in the coordinate domains automatically ensures that the vertex sanity criterion is satisfied.

## Conclusion

We have developed general triangulation criteria for manifolds that are relatively simple to verify for an explicit candidate homeomorphism *H*. Our two main applications are submanifolds of Euclidean space and Riemannian manifolds, for which we improve on the state-of-the-art.

Our conditions are natural and have been used to prove the correctness of triangulation algorithms [8, 16]. Moreover, variants of these conditions may be derived, so as to obtain conditions that are even more convenient to prove the correctness of other algorithms. In a companion paper with André Lieutier [6, Thm. 5] that was motivated by the analysis of a specific algorithm to triangulate point clouds of Euclidean spaces [15], our criteria (1) (compatible atlases) and (3) (vertex sanity) are replaced by the requirement that the projection on local tangent planes of *all* top dimensional simplices inside a ball of small enough radius have disjoint interiors. Note that the requirement involves all the simplices around a vertex, not simply those of the star of a vertex as we assumed in criterion (1). With this stronger condition, one can exploit some specific properties of submanifolds of Euclidean spaces to optimize the constants governing the density of the point sample, obtaining even more relaxed sampling requirements.

## Appendix A: Metric and Differentiable Distortion Maps

In this short section we gather some useful lemmas on general distortion maps, and on differentiable distortion maps.

### A.1 Metric Distortion Maps

The definition of a distortion map (Definition 2.8) makes sense in a more general context: a map $F:(X,d_X) \rightarrow (Y,d_Y)$ between metric spaces is a $\xi$*-distortion map* if

23 $$\begin{aligned} |{d_Y(F(x),F(y)) - d_X(x,y)}| \le \xi d_X(x,y) \quad \ \ \text {for all }\ x,y \in X. \end{aligned}$$

#### Lemma A.1

(inverse and composition of distortion maps)

(i) If $F:(X,d_X) \rightarrow (Y,d_Y)$ is a $\xi$-distortion map with $\xi < 1$, then $F^{-1}$ is a $({\xi }/({1-\xi }))$-distortion map.
(ii) Suppose $F_i:(X_i,d_{X_i})\rightarrow (X_{i+1}, d_{X_{i+1}})$,  $1\le i \le k$, are respectively $\xi _i$-distortion maps. Then
  $$\begin{aligned} F_k \circ F_{k-1} \circ \cdots \circ F_1:X_1 \rightarrow X_{k+1} \end{aligned}$$
  is a $\bigl (\sum _{W \in \wp (\{k\})} \prod _{i\in W} \xi _i\bigr )$-distortion map, where $\wp (\{k\})$ is the set of nonempty subsets of $\{1,\dots , k\}$. In particular, $F_2\circ F_1$ is a $(\xi _1+\xi _2+\xi _1\xi _2)$-distortion map.

#### Proof

(i) Let $u=F(x)$ and $v=F(y)$. Then (23) becomes

24 $$\begin{aligned} |{d_X(F^{-1}(u),F^{-1}(v)) - d_Y(u,v)}| \le \xi d_X(F^{-1}(u),F^{-1}(v)). \end{aligned}$$

But it follows from (24) that $d_X(F^{-1}(u),F^{-1}(v))\le d_Y(u,v)/({1-\xi })$, and plugging this back into (24) yields the result.

(ii) Using the observation that

$$\begin{aligned} d_{X_2}(F_1(x),F_1(y)) \le (1 + \xi _1)d_{X_1}(x,y), \end{aligned}$$

we find

$$\begin{aligned} \begin{aligned}&\bigl |{d_{X_3}(F_2\circ F_1(x), F_2\circ F_1(x)) - d_{X_1}(x,y)}\bigr |\\&\qquad \le \bigl |{ d_{X_3}(F_2\circ F_1(x), F_2\circ F_1(x))- d_{X_2}(F_1(x), F_1(y))}\bigr | \\&\qquad \qquad + \bigl |{d_{X_2}(F_1(x), F_1(y))- d_{X_1}(x,y)}\bigr | \\&\qquad \le \xi _2 d_{X_2}(F_1(x), F_1(y))+ \xi _1 d_{X_1}(x,y) \\&\qquad \le \xi _2 (1 + \xi _1)d_{X_1}(x,y)+ \xi _1 d_{X_1}(x,y)= (\xi _1 +\xi _2 + \xi _2\xi _1)d_{X_1}(x,y). \end{aligned} \end{aligned}$$

This establishes the bound for the composition of two maps, but it also serves as the inductive step for the general bound. If *G* is the $\eta$-distortion map defined by $G=F_{k-1}\circ \cdots \circ F_1$, then $F_k \circ G$ is a $(\xi _k+\eta + \xi _k \eta )$-distortion map. Now notice that

$$\begin{aligned} \sum _{W \in \wp (\{k\})}\prod _{i\in W}\xi _i=\xi _k+\left( \,\sum _{W\in \wp (\{k-1\})}\prod _{i\in W}\xi _i\right) +\xi _k\left( \,\sum _{W\in \wp (\{k-1\})} \prod _{i\in W} \xi _i\right) . \end{aligned}$$

$$\square $$

### A.2 Differentiable Distortion Maps

Although the homeomorphism demonstration that yields Theorem 2.17 makes no explicit requirement of differentiability, it is convenient to exploit differentiability when it is available. We collect here some observations relating metric distortion and bounds on the differential of a map.

Recall that if $F:U\subseteq {\mathbb {R}}^m \rightarrow {\mathbb {R}}^m$ is differentiable, then the *differential* of *F* at *x* is the linear map defined by

$$\begin{aligned} dF_x(v) = \dfrac{d}{dt}F\circ \alpha (t)\big |_{t=0}, \end{aligned}$$

where $\alpha :I\subset {\mathbb {R}}\rightarrow {\mathbb {R}}^m$ is any curve such that $\alpha (0)=x$ and $\alpha '(0) = v$, where $\alpha '$ is the derivative with respect to *t*.

Bounds on the differential of *F* are closely related to the metric distortion of *F*. If $A:U\subseteq {\mathbb {R}}^m \rightarrow {\mathbb {R}}^m$ is a linear map, we let $\Vert A\Vert$ denote the operator norm: $\Vert A\Vert = \sup _{|v|=1}|Av|$. Associated with *A* are *m* nonnegative numbers called the *singular values* of *A*, denoted $s_i(A)$, $1\le i \le m$, ordered such that $s_i(A) \ge s_j(A)$ if $i\le j$. We only mention the singular values because they provide a notational convenience. The largest singular value is defined by $s_1(A)=\Vert A\Vert$, and the smallest singular value is $s_m(A)=\inf _{|v|=1}|Av|$.

#### Lemma A.2

If $F:U \subseteq {\mathbb {R}}^m \rightarrow {\mathbb {R}}^m$ is a differentiable $\xi$-distortion map, then

$$\begin{aligned} |{s_i(dF_x)-1}| \le \xi \ \quad \ \text {for all }\ x\in U\text { and } 1 \le i \le m. \end{aligned}$$

#### Proof

For $x\in U$, and $v \in T_x{\mathbb {R}}^m = {\mathbb {R}}^m$ with $|v|=1$, let $\alpha (t)= x + tv$. Since *F* is a $\xi$-distortion map, we have

$$\begin{aligned} (1-\xi )|(x+tv)-x| \le |{F(x+tv)-F(x)}| \le (1+\xi )|(x+tv)-x|, \end{aligned}$$

so for all $t\ne 0$,

$$\begin{aligned} 1-\xi \le \dfrac{|{F(x+tv) - F(x)}|}{|{t}|} \le 1+\xi . \end{aligned}$$

Since *F* is differentiable,

$$\begin{aligned} \lim _{t\rightarrow 0}\dfrac{|{F(x+tv) - F(x)}|}{|{t}|}= \biggl |\lim _{t\rightarrow 0}\dfrac{F(x+tv) - F(x)}{t}\biggr |= |dF_x(v)|, \end{aligned}$$

so

$$\begin{aligned} 1-\xi \le |dF_x(v)| \le 1+\xi , \end{aligned}$$

which yields the claimed result. $\square$

So a bound on the metric distortion of a differentiable map directly yields the same bound on the amount the singular values of the differential can differ from 1. We are interested in a converse assertion: we want to bound the metric distortion of *F*, given a bound on (the singular values of) its differential. This can only be done with caveats.

#### Lemma A.3

Suppose $\varvec{\omega }$ is a convex set, $\varvec{\omega }\subseteq U \subseteq {\mathbb {R}}^m$, and $F:U \rightarrow {\mathbb {R}}^m$ is a differentiable map such that $F(\varvec{\omega }) \subseteq V \subseteq F(U)$ for some convex set *V*. If *F* is injective, and

$$\begin{aligned} |{s_i(dF_x)-1}| \le \xi < 1,\ \quad \ \text {for all }\ \ x\in U\ \text { and }\ 1 \le i \le m, \end{aligned}$$

then $F|_{\varvec{\omega }}$ is a $\xi$-distortion map.

#### Proof

Since $\varvec{\omega }$ is convex, the length of the image of the line segment between *x* and *y* provides an upper bound on the distance between *F*(*x*) and *F*(*y*), and this length can be bounded because of the bound on *dF*:

25 $$\begin{aligned} |{F(y) - F(x)}| \le \int _0^1 \bigl |dF_{x +t(y-x)}(y-x)\bigr |\, dt\le (1 +\xi )|y-x|. \end{aligned}$$

To get a lower bound we use the fact that injectivity and the bound on the singular values imply that *F* is invertible. For any point $z=F(x)$ we have

$$\begin{aligned} \Vert dF^{-1}_z\Vert = s_m(dF_x)^{-1} \le (1-\xi )^{-1}. \end{aligned}$$

Since, for $x,y\in \varvec{\omega }$ the segment [*F*(*x*), *F*(*y*)] is contained in *F*(*U*), we can use the same argument as in (25), but using $F^{-1}$ instead of *F*, so

$$\begin{aligned} |x-y| \le (1-\xi )^{-1} |F(x)-F(y)|. \end{aligned}$$

Therefore, combining with (25) we have

$$\begin{aligned} (1-\xi )|x-y| \le |F(x)-F(y)|\le (1+\xi ) |x-y|. \end{aligned}$$

$$\square $$

## Appendix B: Elementary Degree Theory

We recall here some basic ideas in degree theory. Our primary motivation is to facilitate the statement and proof of Lemma 2.7, recovering what we need of a result of Whitney [24, Appendix II, Lemma 15a], without using differentiability assumptions.

We are interested in the degree of continuous maps $F:\overline{\varOmega } \rightarrow {\mathbb {R}}^m$, where $\varOmega \subset {\mathbb {R}}^m$ is an open, bounded, and nonempty domain in ${\mathbb {R}}^m$, as can be found in Chapter IV, Sects. 1 and 2 of [22], for example. As with most modern treatments of degree theory, Outerelo and Ruiz start by defining the degree for a regular value of a differentiable map, where the idea is transparent. For $y \in {\mathbb {R}}^m \setminus F(\partial {\varOmega })$, the degree is defined as

$$\begin{aligned} \deg (F,\varOmega ,y)\, :=\! \sum _{x\in F^{-1}(y)}\!{{\,\mathrm{sgn}\,}}_x(F), \end{aligned}$$

where ${{\,\mathrm{sgn}\,}}_x(F)$ denotes the sign ($\pm 1$) of the Jacobian determinant (the determinant of the differential of *F*) at *x*. Thus the degree at *y* counts the number of points in the preimage, accounting for the local orientation of the map.

It is then shown that the degree is locally constant on the (open) set of regular values, and after showing that it is also invariant under homotopies that avoid conflicts between *y* and the image of the boundary, the degree is defined for an arbitrary point in ${\mathbb {R}}^m \setminus F(\partial {\varOmega })$, and it is constant on each connected component of ${\mathbb {R}}^m \setminus F(\partial {\varOmega })$.

Then the definition of degree is extended to continuous maps $F:\overline{\varOmega } \rightarrow {\mathbb {R}}^m$ by means of the Weierstrass approximation theorem (here $\Vert \,\cdot \,\Vert _{\infty }$ denotes the supremum norm):

### Lemma B.1

[22, Prop. and Defn. IV.2.1] Let $F:\overline{\varOmega } \rightarrow {\mathbb {R}}^m$ be a continuous map, and let $y\in {\mathbb {R}}^m \setminus F(\partial {\varOmega })$. Then there exists a smooth map $G:\overline{\varOmega } \rightarrow {\mathbb {R}}^m$ such that $\Vert F-G\Vert _{\infty } <d_{{\mathbb {R}}^m}(y,F(\partial {\varOmega }))$. For all such *G*, the degree $\deg (G,\varOmega ,y)$ is defined ($y\in {\mathbb {R}}^m\setminus G(\partial {\varOmega })$) and is the same, and we define the degree of *F* by

$$\begin{aligned} \deg (F,\varOmega ,y)=\deg (G,\varOmega ,y). \end{aligned}$$

Furthermore, *G* can be chosen such that *y* is a regular value of $G|_{\varOmega }$, and then

$$\begin{aligned} \deg (F,\varOmega ,a) \,=\! \sum _{x\in G^{-1}(y)} \!{{\,\mathrm{sgn}\,}}_x(G). \end{aligned}$$

The locally constant nature of the degree is the main property we wish to exploit:

### Lemma B.2

[22, Prop. IV.2.3] Let $F:\overline{\varOmega } \rightarrow {\mathbb {R}}^m$ be a continuous map. Then the degree $y \mapsto \deg (F,\varOmega ,y)$ is constant on every connected component of ${\mathbb {R}}^m \setminus F(\partial {\varOmega })$.

Notice that if *F* is a topological embedding, then $\deg (F,\varOmega ,y) = \pm 1$ for any point $y \in F(\varOmega )$. We will also have occasion to use the following:

### Lemma B.3

[22, Cor. IV.2.5 (3)] Given a continuous mapping $F :\overline{\varOmega } \rightarrow {\mathbb {R}}^m$, two disjoint open subsets $\varOmega _1, \varOmega _2 \subset \varOmega$, and a point $y\notin F(\overline{\varOmega }\setminus (\varOmega _1 \cap \varOmega _2))$,

$$\begin{aligned} \deg (F,\varOmega ,y) = \deg (F,\varOmega _1,y) + \deg (F,\varOmega _2, y). \end{aligned}$$

Since no connectedness assumptions are made on the open sets in question, a straightforward inductive argument allows us to strengthen the statement of Lemma B.3:

### Lemma B.4

Suppose $\varOmega _1, \ldots , \varOmega _n$ are mutually disjoint open subsets of the open domain $\varOmega \subset {\mathbb {R}}^m$, and $F:\overline{\varOmega } \rightarrow {\mathbb {R}}^m$ is a continuous map. If $y \notin F\bigl (\overline{\varOmega } \setminus \bigcup _{i=1}^n\varOmega _i\bigr )$, then

$$\begin{aligned} \deg (F,\varOmega ,y) = \sum _{i=1}^n \deg (F,\varOmega _i,y). \end{aligned}$$

### B.1 Orientation and Cogent Maps

A simplex is *oriented* by choosing an orientation for its affine hull, or equivalently, by ordering its vertices; any even permutation of this order describes the same orientation. An *m*-simplex in ${\mathbb {R}}^m$ has a natural orientation induced from the canonical orientation of ${\mathbb {R}}^m$ defined by the standard basis. Our convention is that $\varvec{\sigma }\subset {\mathbb {R}}^m$ is positively oriented if its vertices $v_i$, $0\le i\le m$, are ordered such that the basis $\{v_1-v_0, \ldots ,v_m-v_0\}$ defines the same orientation as the canonical basis of ${\mathbb {R}}^m$.

In the case that concerns us, where ${\mathscr {C}}$ is a finite pure *m*-complex piecewise linearly embedded in ${\mathbb {R}}^m$ (so that we can naturally view $|{\mathscr {C}}|\subset {\mathbb {R}}^m$), we assume that the *m*-simplices carry the canonical orientation inherited from the ambient space.

#### Definition B.5

(*orientation preserving map*)   If $\varvec{\sigma }\subset {\mathbb {R}}^m$ is an *m*-simplex, we say that a continuous topological embedding $F:\varvec{\sigma }\rightarrow {\mathbb {R}}^m$ is *orientation preserving*, or *positive*, if $\deg (F,{{\,\mathrm{relint}\,}}(\varvec{\sigma }),y)=1$, for any point $y\in {{\,\mathrm{int}\,}}(F(\varvec{\sigma }))$, otherwise *F* is *orientation reversing*.

#### Definition B.6

(*cogent maps*) Suppose ${\mathscr {C}}$ is a pure *m*-complex. We call a map $F:|{\mathscr {C}}| \rightarrow {\mathbb {R}}^m$ *cogent* with respect to ${\mathscr {C}}$ if it is continuous and its restriction to each simplex is an embedding.

For cogent maps, the points in ${\mathbb {R}}^m \setminus F(|{\mathscr {C}}^{m-1}|)$ are analogous to the regular values of a differentiable map. If *F* is a cogent map and $x\in {{\,\mathrm{relint}\,}}(\varvec{\sigma })$, where $\varvec{\sigma }$ is an *m*-simplex in ${\mathscr {C}}$, we define

26 $$\begin{aligned} {{\,\mathrm{sgn}\,}}_x(F) := \deg (F,{{\,\mathrm{relint}\,}}(\varvec{\sigma }), F(x)). \end{aligned}$$

This makes the analogy with the degree of a differentiable map transparent:

#### Lemma B.7

(degree of cogent maps)   Suppose ${\mathscr {C}}$ is a finite pure *m*-complex embedded in ${\mathbb {R}}^m$. Let $\varOmega = |{\mathscr {C}}| \setminus \partial {|{\mathscr {C}}|}$. If a continuous map $F:|{\mathscr {C}}| \rightarrow {\mathbb {R}}^m$ is cogent with respect to ${\mathscr {C}}$, then for any $y\in {\mathbb {R}}^m\setminus F(|{\mathscr {C}}^{m-1}|)$ (recall that $\partial {|{\mathscr {C}}|}\subseteq |{\mathscr {C}}^{m-1}|$ [4, Lemmas 3.6 and 3.7]),

$$\begin{aligned} \deg (F,\varOmega ,y) = \sum _{x \in F^{-1}(y)} {{\,\mathrm{sgn}\,}}_x(F). \end{aligned}$$

A very minor modification to the proof of Lemma B.1 would allow us to sharpen the bound on $\Vert F-G\Vert _{\infty }$ in our context, so that

$$\begin{aligned} \Vert F-G\Vert _{\infty } <\min _{\varvec{\sigma }\cap F^{-1}(y)\ne \emptyset }d_{{\mathbb {R}}^m}(y,F(\partial {\varvec{\sigma }})), \end{aligned}$$

and Lemma B.7 follows immediately from the same proof. But rather than digging into the proof of that lemma, we can avoid getting our hands dirty and just exploit Lemma B.4.

#### Proof of Lemma B.7

The preimage of *y* is a finite set of points: $F^{-1}(y) = \{x_i\}$, $i\in \{1,\ldots ,n\}$. For each *i*, let $\varvec{\sigma }_i$ be the *m*-simplex that contains $x_i$ in its interior, and set $\varOmega _i = {{\,\mathrm{relint}\,}}(\varvec{\sigma }_i)$. Then the statement follows from Lemma B.4 and the definition (26) of ${{\,\mathrm{sgn}\,}}_{x_i}(F)$. $\square$

#### Definition B.8

(*simplexwise positive*)   A map is *simplexwise positive* if it is cogent and its restriction to any *m*-simplex is orientation preserving.

Lemmas B.2 and B.7 yield

#### Lemma B.9

If ${\mathscr {C}}$ is a finite pure *m*-complex embedded in ${\mathbb {R}}^m$, and $F :|{\mathscr {C}}| \rightarrow {\mathbb {R}}^m$ is simplexwise positive, then for any connected open subset *W* of ${\mathbb {R}}^m \setminus F(\partial {|{\mathscr {C}}|})$, any two points of *W* not in $F(|{\mathscr {C}}^{m-1}|)$ are covered the same number of times (i.e., have the same number of points in their preimage under *F*).

#### Remark B.10

Whitney’s Lemma [24, Appx. II, Lem. 15] is a combination of Lemmas B.9 and 2.7, but applies in more generality. However, he assumes that the restriction of *F* to each *m*-simplex is a smooth map. The generality can be fully recovered without invoking this differentiability assumption.

The definition of the degree of a map $\overline{\varOmega } \rightarrow {\mathbb {R}}^m$ can be naturally extended to the case where $\overline{\varOmega }$ is an oriented abstract manifold with boundary. Using this, the assumption that ${\mathscr {C}}$ is embedded in ${\mathbb {R}}^m$ can be dropped. Also, the assumption that ${\mathscr {C}}$ be finite can be relaxed, at least if we assume that *F* is proper (so that the number of points in the preimage of a point is finite—Whitney seems to assume this). Whitney also only assumed that ${\mathscr {C}}$ was a *pseudomanifold* with boundary: a pure *m*-complex such that any $(m-1)$-simplex is a face of either two or one *m*-simplices (those that are the face of only one *m*-simplex define the boundary complex).

Brouwer’s original exposition of degree theory [12] used simplicial approximations, and piecewise linear maps, rather than differentiable maps as the foundation. Even though the simplicial aspect is attractive and natural in our setting, there would be no economy in using this approach for our purposes. However, Brouwer’s exposition was based on the notion of pseudomanifolds [22, p. 23], so that approach would also allow us to recover the pseudomanifold aspect of Whitney’s lemma.

## References

[1] Amenta N, Bern M (1999). Surface reconstruction by Voronoi filtering. Discrete Comput. Geom..

[2] Amenta N, Choi S, Dey TK, Leekha N (2002). A simple algorithm for homeomorphic surface reconstruction. Int. J. Comput. Geom. Appl..

[3] Boissonnat J-D, Chazal F, Yvinec M (2018). Geometric and Topological Inference. Cambridge Texts in Applied Mathematics.

[4] Boissonnat J-D, Dyer R, Ghosh A (2013). The stability of Delaunay triangulations. Int. J. Comput. Geom. Appl..

[5] Boissonnat J-D, Dyer R, Ghosh A (2018). Delaunay triangulation of manifolds. Found. Comput. Math..

[6] Boissonnat J-D, Dyer R, Ghosh A, Lieutier A, Wintraecken M (2021). Local conditions for triangulating submanifolds of Euclidean space. Discrete Comput. Geom..

[7] Boissonnat, J., Dyer, R., Ghosh, A., Wintraecken, M.: Local criteria for triangulation of manifolds (2018). arXiv:1803.07642 10.1007/s00454-022-00431-7 PMC9805998 36605029

[8] Boissonnat J-D, Ghosh A (2014). Manifold reconstruction using tangential Delaunay complexes. Discrete Comput. Geom..

[9] Boissonnat J-D, Kachanovich S, Wintraecken M (2021). Triangulating submanifolds: an elementary and quantified version of Whitney’s method. Discrete Comput. Geom..

[10] Boissonnat J-D, Lieutier A, Wintraecken M (2019). The reach, metric distortion, geodesic convexity and the variation of tangent spaces. J. Appl. Comput. Topol..

[11] Boissonnat J-D, Oudot S (2005). Provably good sampling and meshing of surfaces. Graph. Models.

[12] Brouwer LEJ (1912). Über Abbildung von Mannigfaltigkeiten. Math. Ann..

[13] Cairns SS (1934). On the triangulation of regular loci. Ann. Math..

[14] Cheng, S.-W., Dey, T.K., Ramos, E.A.: Manifold reconstruction from point samples. In: 16th Annual ACM-SIAM Symposium on Discrete Algorithms (Vancouver 2005), pp. 1018–1027. ACM, New York (2005)

[15] Cohen-Steiner, D., Lieutier, A., Vuillamy, J.: Lexicographic optimal homologous chains and applications to point cloud triangulations. In: 36th International Symposium on Computational Geometry. Leibniz Int. Proc. Inform., vol. 164, # 32. Leibniz-Zent. Inform., Wadern (2020)

[16] Dyer R, Vegter G, Wintraecken M (2015). Riemannian simplices and triangulations. Geom. Dedicata.

[17] Dyer R, Zhang H, Möller T (2008). Surface sampling and the intrinsic Voronoi diagram. Comput. Graph. Forum.

[18] Edelsbrunner H, Shah NR (1997). Triangulating topological spaces. Int. J. Comput. Geom. Appl..

[19] Federer H (1959). Curvature measures. Trans. Am. Math. Soc..

[20] Lundell AT, Weingram S (1969). The Topology of CW Complexes. The University Series in Higher Mathematics.

[21] Munkres JR (1968). Elementary Differential Topology.

[22] Outerelo E, Ruiz JM (2009). Mapping Degree Theory.

[23] Whitehead JHC (1940). On $$C^1$$-complexes. Ann. Math..

[24] Whitney H (1957). Geometric Integration Theory.

